# A cyclic pyrrole-imidazole polyamide reduces pathogenic RNA in CAG/CTG triplet repeat neurological disease models

**DOI:** 10.1172/JCI164792

**Published:** 2023-11-15

**Authors:** Susumu Ikenoshita, Kazuya Matsuo, Yasushi Yabuki, Kosuke Kawakubo, Sefan Asamitsu, Karin Hori, Shingo Usuki, Yuki Hirose, Toshikazu Bando, Kimi Araki, Mitsuharu Ueda, Hiroshi Sugiyama, Norifumi Shioda

**Affiliations:** 1 Department of Genomic Neurology, Institute of Molecular Embryology and Genetics (IMEG),; 2 Department of Neurology, Graduate School of Medical Sciences,; 3 Graduate School of Pharmaceutical Sciences, and; 4 Liaison Laboratory Research Promotion Center, IMEG, Kumamoto University, Kumamoto, Japan.; 5 Department of Chemistry, Graduate School of Science, Kyoto University, Kyoto, Japan.; 6 Institute of Resource Development and Analysis and; 7 Center for Metabolic Regulation of Healthy Aging, Kumamoto University, Kumamoto, Japan.; 8 Institute for Integrated Cell-Material Science (iCeMS), Kyoto University, Kyoto, Japan.

**Keywords:** Neuroscience, Therapeutics, Genetic diseases, Noncoding RNAs, Transcription

## Abstract

Expansion of CAG and CTG (CWG) triplet repeats causes several inherited neurological diseases. The CWG repeat diseases are thought to involve complex pathogenic mechanisms through expanded CWG repeat–derived RNAs in a noncoding region and polypeptides in a coding region, respectively. However, an effective therapeutic approach has not been established for the CWG repeat diseases. Here, we show that a CWG repeat DNA–targeting compound, cyclic pyrrole–imidazole polyamide (CWG-cPIP), suppressed the pathogenesis of coding and noncoding CWG repeat diseases. CWG-cPIP bound to the hairpin form of mismatched CWG DNA, interfering with transcription elongation by RNA polymerase through a preferential activity toward repeat-expanded DNA. We found that CWG-cPIP selectively inhibited pathogenic mRNA transcripts from expanded CWG repeats, reducing CUG RNA foci and polyglutamine accumulation in cells from patients with myotonic dystrophy type 1 (DM1) and Huntington’s disease (HD). Treatment with CWG-cPIP ameliorated behavioral deficits in adeno-associated virus–mediated CWG repeat–expressing mice and in a genetic mouse model of HD, without cytotoxicity or off-target effects. Together, we present a candidate compound that targets expanded CWG repeat DNA independently of its genomic location and reduces both pathogenic RNA and protein levels. CWG-cPIP may be used for the treatment of CWG repeat diseases and improvement of clinical outcomes.

## Introduction

Short tandem repeats (STRs), also known as microsatellites, are polymorphic repeat sequences with 1–6 bp motifs scattered throughout the human genome (1). STRs are highly unstable in a repeat length–dependent manner, and the expansion of repeat length across generations results in diseases that primarily affect the central nervous system (2, 3). In particular, the expansion of CAG and CTG (CWG) triplet repeats cause many neurological diseases. These repeats can be classified into the following 2 types according to their genomic location: (a) CAG repeat expansion in coding regions; for example, in Huntington’s disease (HD); spinocerebellar ataxia (SCA) types 1, 2, 3, 6, 7, and 17; spinal and bulbar muscular atrophy; and dentatorubral pallidoluysian atrophy and (b) CTG repeat expansion in noncoding regions, especially the 3′-UTRs; for example, in myotonic dystrophy type 1 (DM1) and SCA8 (2–5). While CAG repeat diseases in coding regions typically change the repeat tract size by 10 or fewer units per generation, CTG repeat diseases in noncoding regions increase by 100 to 10,000 units per generation (6, 7).

The mechanisms by which expanded CAG repeats in coding genes contribute to disease pathogenesis have been extensively discussed at DNA, RNA, and polyglutamine (polyQ) levels. Translated polyQ tracts form amyloid cores, initiating protein misfolding and aggregation that ultimately leads to neurodegeneration (8, 9). As causative genes with CAG repeat expansion have no sequence homology or functional similarity (2), expanded polyQ tracts are implicated as causal factors in CAG repeat diseases of coding regions. At the RNA level, the interruption of penultimate CAA within the glutamine-encoding sequence is closely linked to the timing of HD onset, and the mutation with loss of the CAA codon accelerates the onset, regardless of the polyQ tract length (10–12). In addition, the CAA interruption thermodynamically destabilizes the hairpin-structured RNA transcripts from the CAG tract in SCA1 and SCA2 (13), suggesting a link between RNA secondary structures and the pathogenesis of polyQ diseases. At the DNA level, some genes involved in DNA maintenance, such as *MLH1* and *PMS2*, are implicated as rate determinants for the onset of HD by modifying the somatic expansion of CAG repeat DNA (11, 14).

CTG repeat expansion diseases in noncoding regions are mainly driven by RNA toxicity (5). DM1 is caused by a CTG repeat expansion in the 3′-UTR of *DMPK* and is the most common neuromuscular disorder (15–17). DM1 (OMIM #160900) is characterized by myotonia, muscle weakness, and cognitive dysfunction. CUG RNA transcribed from the expanded CTG repeats adopts a highly stable mismatched hairpin structure that forms nuclear RNA foci (18–20). Although the toxic mechanism of nuclear RNA foci remains unclear, CUG RNA–binding proteins such as the muscleblind-like (MBNL) family are sequestered, and CUG-binding protein 1 (CUGBP1) is upregulated by nuclear RNA foci, triggering aberrant alternative splicing of specific pre-mRNAs (21, 22).

In addition to the pathogenic mechanisms of polyQ toxicity and RNA toxicity, the CWG repeats expansion may also induce cell death indirectly by repeat-associated non-AUG (RAN) translation into toxic polypeptides (23, 24). RAN translation was first reported in the noncoding CTG repeat diseases DM1 and SCA8 (23) and has also been found in some coding CAG repeat diseases, including HD (24, 25).

CWG repeat diseases are thought to be caused by highly complex intracellular mechanisms, and no effective treatment has been developed to date. Antisense oligonucleotides (ASOs) that eliminate pathogenic repeat RNAs have been developed. However, a series of clinical trials using ASOs have been terminated (26–29). To address this issue, we focused on the transcription inhibition of CWG repeat DNA as a therapeutic target. Here, we assessed the potential of a CWG triplet repeat DNA-targeting compound cyclic pyrrole–imidazole polyamide (CWG-cPIP) to inhibit expanded CWG repeat–derived mRNA transcription in DM1- and HD-derived human cells, as well as to control neuronal dysfunction in adeno-associated virus–mediated (AAV-mediated) CWG repeat–expressing mice and a genetic mouse model of HD.

## Results

### CWG-cPIP binds preferentially to repeat-expanded CWG DNA.

**Figure 1:**
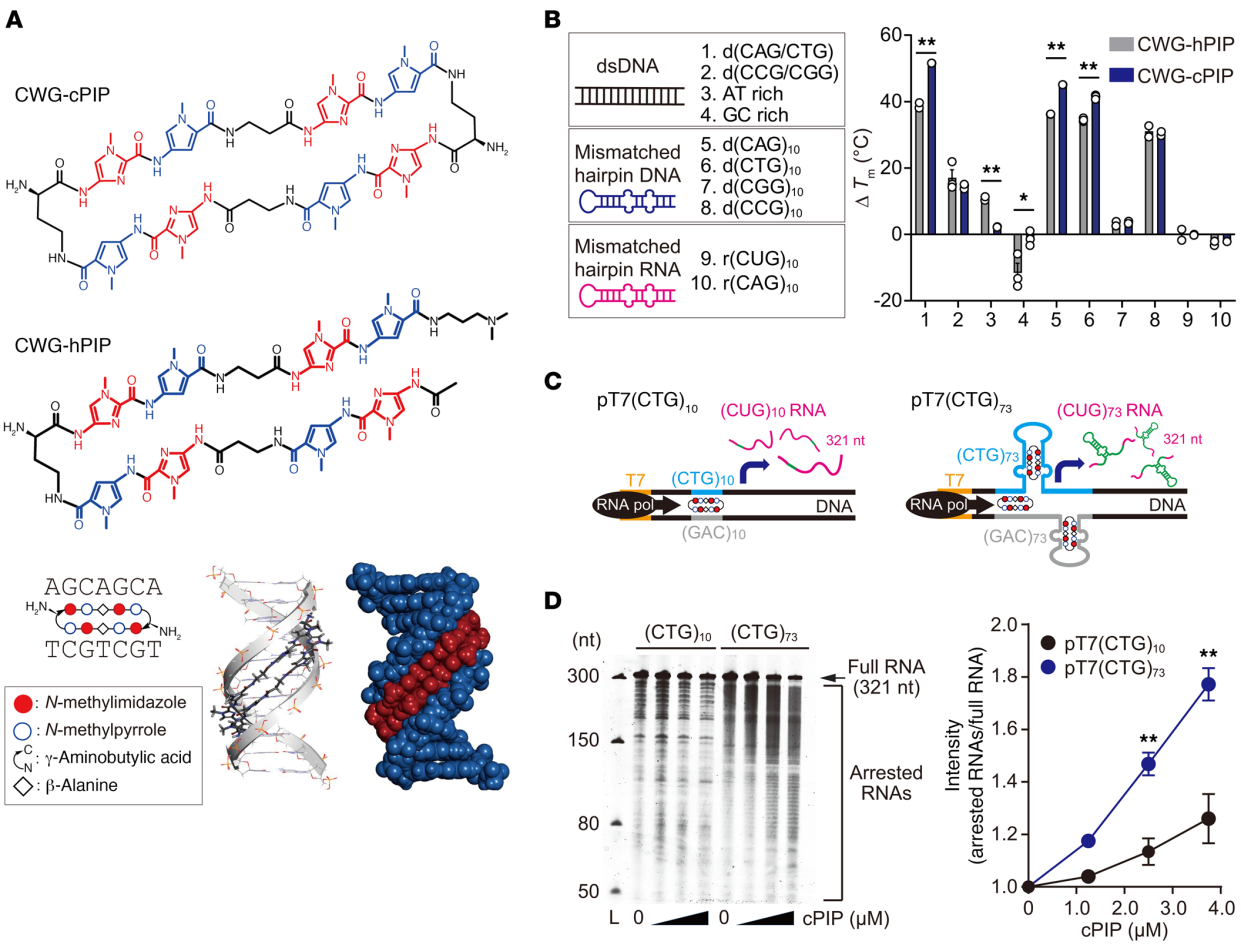
Transcriptional inhibition of CWG repeat DNA by CWG-cPIP. (**A**) Chemical structure of CWG-cPIP and CWG-hPIP; a schematic illustration of DNA sequence recognition of CWG-cPIP (bottom left); and molecular models of CWG-cPIP/double-stranded CWG-DNA complex by computer-assisted molecular simulation. (**B**) Nucleic acid sequences used for the *T*_m_ assay and quantification of Δ*T*_m_. The number on the *x* axis corresponds to the nucleic acid sequence on the left legend. *P < 0.05 and **P < 0.01, by 2-sided, unpaired Student’s *t* test. *n* = 2 [1. d(CAG/CTG); 3. AT rich; 5. d(CAG)_10_; 7. d(CGG)_10_; 9. r(CUG)_10_; 10. r(CAG)_10_]; *n* = 3 [2. d(CCG/CGG); 4. GC rich; 6. d(CTG)_10_; 8. d(CCG)_10_]. (**C**) Schematic representation of the in vitro transcription arrest assay. (**D**) Representative urea polyacrylamide gel electrophoresis for the in vitro transcription arrest assay (left). CWG-cPIP concentrations were 1.25, 2.5, and 3.75 μM. The arrow and bracket represent transcribed full-length RNAs and arrested-form RNAs, respectively. Graph on the right shows quantification of the arrested RNAs. **P < 0.01, by 2-way ANOVA with Bonferroni’s multiple-comparison test. *n* = 3 each. L, ladder; nt, nucleotide. Data represent the mean ± SEM. Statistical data are provided in Supplemental Data File 6.

PIPs are composed of amide-linked *N*-methyl pyrrole (Py) and *N*-methyl imidazole (Im) residues. PIPs can be optimized and synthesized to target DNA sequences and bind noncovalently to DNA minor grooves in a sequence-specific manner. Im/Py pairs recognize G/C base pairs, whereas Py/Py, β-alanine, and γ-turn pairs recognize A/T and T/A bp (30, 31). We have previously developed many types of PIPs with sequence specificity, including anticancer agents (32), DNA fluorescence probes (33), and gene regulators (34, 35). In addition, we recently found that cyclic-type PIPs (cPIPs) with 2 γ-turn units showed higher DNA sequence selectivity and binding affinity than did traditional hairpin-type PIPs (hPIPs) (36). On the basis of these chemical discoveries, we developed a CWG-cPIP for CWG repeat diseases (Figure 1A) (37).

To investigate the selectivity and binding affinity of CWG-cPIP to the target DNA sequence, we conducted a melting temperature (*T*_m_) assay, wherein Δ*T*_m_ was measured for several sequences (Δ*T*_m_ = *T*_m_ [DNA or RNA + PIP] – *T*_m_ [DNA or RNA]). CWG-cPIP bound to double-stranded CWG DNA but not to AT-rich or GC-rich double-stranded DNA with high specificity (Figure 1B and Supplemental Table 1; supplemental material available online with this article; https://doi.org/10.1172/JCI164792DS1). To further investigate the binding properties of CWG-cPIP, we performed a *T*_m_ assay using 5′-(CAG)_10_-3′ and 5′-(CTG)_10_-3′ repeat DNAs containing 3 A/A and T/T mismatched pairs, respectively. CWG-cPIP also showed a high binding affinity for these CWG-mismatched repeat DNAs Figure 1B) (37). Furthermore, we confirmed that CWG-cPIP does not bind to CWG repeat RNA. Importantly, CWG-cPIP showed a significantly higher binding affinity than did a traditional CWG-hPIP for CWG repeat DNA in both double-stranded and mismatched structures (38). Unexpectedly, both CWG-cPIP and CWG-hPIP showed high affinity for the 5′-(CCG)_10_-3′ repeat DNA (Figure 1B and Supplemental Table 1). To elucidate the underlying cause of this phenomenon, we performed molecular modeling studies of CWG-cPIP binding to repeat DNA (Supplemental Figure 1). We found that CCG repeat DNA interacted with CWG-cPIP at the same proximal distance as CWG repeat DNA, suggesting that high affinity for CCG repeat DNA is a common characteristic of PIPs targeting CWG repeat DNA.

PIPs are known to stably interfere with transcription elongation by RNA polymerase II (pol II) for more than 20 hours in vitro (39). During transcription elongation, pol II recognizes PIPs bound to DNA through its own Switch 1 region and is arrested 2–5 bp upstream of the site (39). To investigate the inhibitory effect of CWG-cPIP on transcription elongation by pol II, we performed an in vitro transcription arrest assay using CTG repeat DNA containing the normal range (CTG)_10_ or the pathogenic range (CTG)_73_, which produces 321 nt RNA under the T7 promoter (Figure 1C). As the concentration of CWG-cPIP increased, the amount of transcribed full-length RNA decreased (arrow, 321 nucleotide), and multiple arrested RNAs accumulated (bracket) (Figure 1D). Quantitative evaluation showed that CWG-cPIP treatment produced significantly more arrested products from (CTG)_73_ DNA than from (CTG)_10_ DNA (Figure 1D). These results suggested that CWG-cPIP inhibited pol II transcription elongation by preferentially targeting repeat-expanded DNA rather than normal repeat DNA.

### CWG-cPIP inhibits the production of pathogenic CUG RNA in DM1 and polyQ in HD cells.

**Figure 2:**
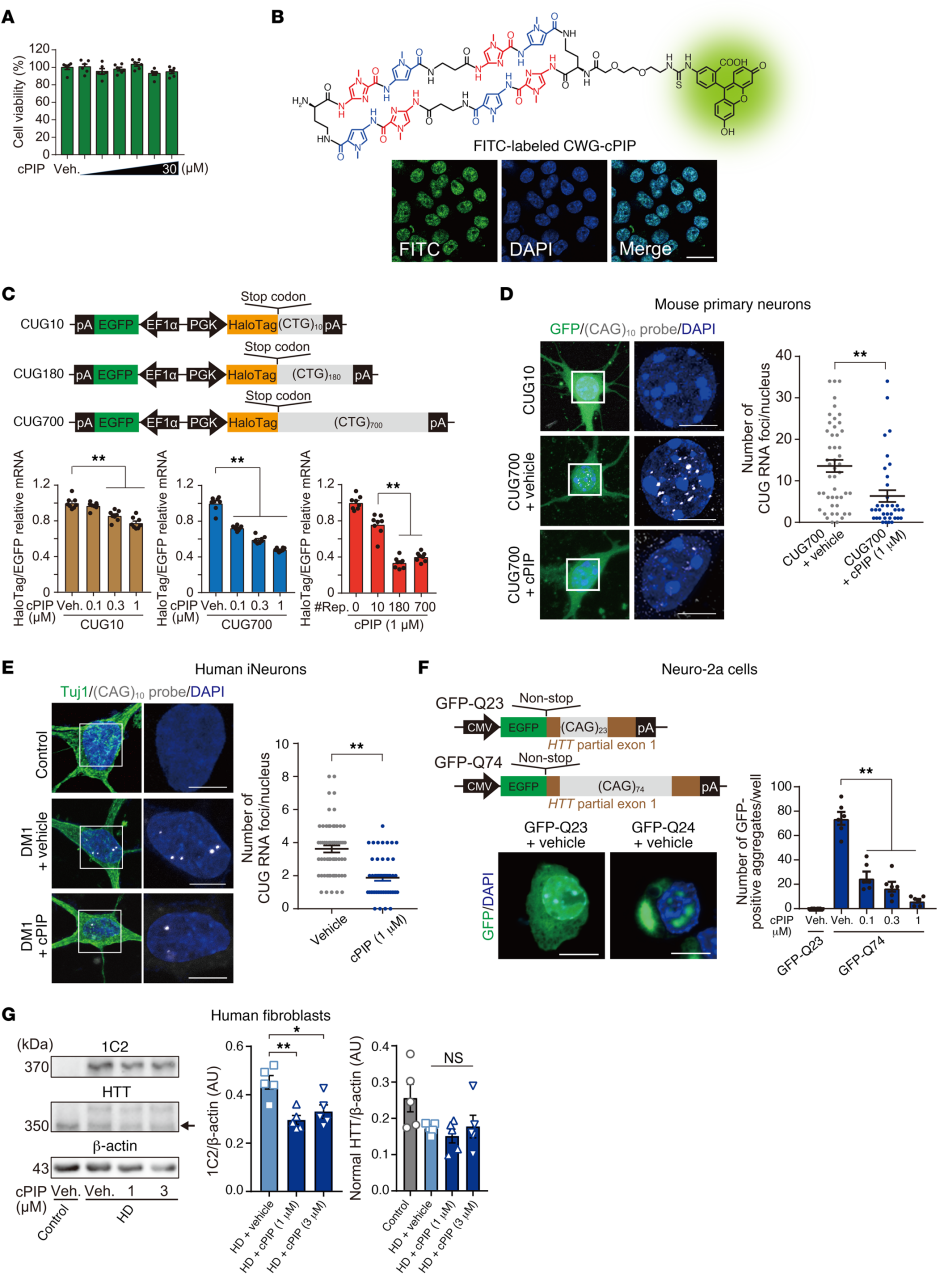
Attenuation of pathogenic CUG RNA foci and polyQ aggregates in DM1 and HD cell models by CWG-cPIP treatment. (**A**) Cell viability assay in Neuro-2a cells treated with CWG-cPIP at concentrations of 0.1, 0.3, 1, 3, 10, and 30 μM. Statistics were performed by 1-way ANOVA with Bonferroni’s multiple-comparison test. *n* = 6 each. (**B**) Chemical structure of FITC-labeled CWG-cPIP and representative confocal images of FITC-labeled CWG-cPIP. Nuclei were counterstained with DAPI (blue). Scale bar: 20 μm. (**C**) Schematic representation of constructs used for RT-qPCR in cellulo and quantification of HaloTag mRNA levels. **P < 0.01, by 1-way ANOVA with Bonferroni’s multiple-comparison test. *n* = 8 each. #Rep., CUG repeat lengths. (**D**) Representative confocal images of CUG-RNA foci (white) in mouse primary neurons (scale bars: 5 μm) and quantification of CUG-RNA foci (right). **P < 0.01, by 2-sided, unpaired Student’s *t* test. CUG700 plus vehicle: *n* = 49 cells; CUG700 plus CWG-cPIP: *n* = 36 cells. (**E**) Representative confocal images of CUG-RNA foci (white) in DM1 patient–derived iNeurons (scale bars: 5 μm) and quantification of CUG-RNA foci. **P < 0.01, by 2-sided, unpaired Student’s *t* test. Vehicle: *n* = 61 cells; CWG-cPIP: *n* = 49 cells. (**F**) Schematic representation of constructs containing *Egfp* tagged with CAG repeat sequences in a coding region and representative confocal images of GFP-positive aggregates in Neuro-2a cells. Scale bars: 10 μm. Graph shows quantification of GFP-positive aggregates. **P < 0.01, by 1-way ANOVA with Bonferroni’s multiple-comparison test. *n* = 6 wells each. (**G**) Representative blots of lysates from HD patient–derived fibroblasts probed with 1C2 and HTT antibodies. Arrow indicates HTT products corresponding to the normal allele. Graphs show quantification of 1C2 and HTT. *P < 0.05 and **P < 0.01, by 1-way ANOVA with Bonferroni’s multiple-comparison test. *n* = 5 experiments each. Data represent the mean ± SEM. Statistical data are provided in Supplemental Data File 6. Veh., vehicle treatment.

We examined whether CWG-cPIP is effective in cells with pathogenic CWG repeat DNA sequences. First, we investigated the cytotoxicity of CWG-cPIP in intact Neuro-2a cells using a cell viability assay. We found that CWG-cPIP, even at a concentration of 30 μM for 48 hours, had no significant impact on cell viability (Figure 2A). To investigate cell membrane permeability and intracellular residence duration of CWG-cPIP, we synthesized FITC-labeled CWG-cPIP (Supplemental Figure 2) and administered it to intact Neuro-2a cells. We observed FITC-labeled CWG-cPIP (1 μM) in cell nuclei using confocal microscopy for more than 3 days without drug delivery systems (DDSs) such as liposomes (Figure 2B). To assess the off-target effects of CWG-cPIP on gene expression, we performed RNA-Seq analysis of RNAs extracted from the control fibroblasts along with spike-in control RNAs (40) 7 days after treatment with CWG-cPIP (1 μM). Based on a cutoff of an adjusted *P* value of less than 0.05 and a |log_2_ fold change| of greater than 0.5, we observed no changes in gene expression levels following the treatment, suggesting that CWG-cPIP had no significant effect on global transcription (Supplemental Figure 3A and Supplemental Data File 1).

Next, we examined the effect of CWG-cPIP on the production of pathogenic CUG RNA in Neuro-2a cells transfected with a plasmid expressing (CUG)_10_, (CUG)_180_, or (CUG)_700_ repeats in the 3′-UTR of HaloTag mRNA. To normalize transfection efficiency, we used a dual-promoter vector expressing 2 different genes: HaloTag with CTG repeats and *Egfp* as an internal reference (Figure 2C). We observed that HaloTag-(CUG)_700_ mRNA expression was significantly decreased 12 hours after CWG-cPIP treatment at concentrations as low as 0.1 μM and over 50% at 1 μM compared with that after vehicle treatment. In contrast, HaloTag-(CUG)_10_ mRNA expression was suppressed by only 20%, even at the highest concentration of 1 μM CWG-cPIP, compared with expression levels after vehicle treatment. Thus, treatment with CWG-cPIP considerably suppressed the expression of HaloTag-CUG mRNA with expanded repeats (Figure 2C). Compared with expression levels after vehicle treatment, treatment with 3 μM CWG-hPIP suppressed HaloTag-(CUG)_700_ mRNA expression by approximately 15% (Supplemental Figure 4A). In primary mouse cortical neurons transfected with a plasmid expressing (CUG)_10_ or (CUG)_700_ repeats in the 3′-UTR of *Egfp* mRNA, treatment with CWG-cPIP (1 μM for 14 days) considerably suppressed the expression of EGFP-(CUG)_700_ mRNA but not EGFP-(CUG)_10_ mRNA (Supplemental Figure 4B).

Next, we performed FISH with a Cy5-labeled (CAG)_10_ repeat probe to detect CUG RNA foci in mouse primary neurons transfected with a plasmid expressing (CUG)_10_ or (CUG)_700_ repeats in the 3′-UTR of *Egfp* mRNA. EGFP-(CUG)_10_ mRNA–expressing neurons had no detectable CUG RNA foci, whereas EGFP-(CUG)_700_ mRNA–expressing neurons remarkably accumulated nuclear CUG RNA foci. The number of nuclear CUG RNA foci was significantly decreased following CWG-cPIP treatment at 1 μM for 14 days (Figure 2D). We examined the inhibitory effect of CWG-cPIP on endogenous CTG repeat–derived CUG RNA foci using DM1 patient–derived fibroblasts and induced neurons (iNeurons). Treatment with 1 μM CWG-cPIP for 3 days significantly reduced the number of nuclear CUG RNA foci in DM1 fibroblasts and iNeurons (Figure 2E and Supplemental Figure 4C).

We further examined whether CWG-cPIP inhibits pathogenic mRNA derived from coding gene expansion. Neuro-2a cells were transfected with a plasmid expressing HaloTag mRNA tagged with a (CAG)_23_ or (CAG)_74_ repeat sequence within a part of exon 1 of the *HTT* gene (Supplemental Figure 4D) and treated with CWG-cPIP for 12 hours. CWG-cPIP effectively suppressed HaloTag-(CAG)_74_ mRNA expression but not HaloTag-(CAG)_23_ mRNA expression at a lower concentration (Supplemental Figure 4D).

We also assessed whether treatment with CWG-cPIP suppresses polyQ inclusion body formation in Neuro-2a cells transfected with a plasmid expressing *Egfp* tagged with a (CAG)_23_ or (CAG)_74_ repeat sequence within exon 1 of the *HTT* gene, termed EGFP-Q23 and EGFP-Q74, respectively. We observed EGFP-positive aggregates of various sizes in the nuclei and cytoplasm of EGFP-Q74–expressing cells but not in EGFP-Q23–expressing cells, and EGFP-positive aggregates were significantly reduced by CWG-cPIP treatment (Figure 2F). The levels of polyQ-expanded huntingtin (HTT) protein detected by an anti-polyQ tract antibody (clone 1C2) markedly decreased following CWG-cPIP treatment in HD patient–derived fibroblasts compared with their levels in vehicle-treated fibroblasts. Importantly, there were no changes in normal HTT protein levels in HD fibroblasts following CWG-cPIP treatment (Figure 2G).

### Treatment with CWG-cPIP ameliorates cognitive deficit in AAV-mediated CWG repeat–expressing mice.

We assessed the potential of CWG-cPIP in inhibiting the production of pathogenic CUG RNA foci and polyQ in vivo and restoring CWG repeat disease–mediated changes at the behavioral, physiological, and molecular levels. Intravenously administered PIPs could not be detected in the mouse brain by PET imaging (41), suggesting that there was little brain translocation of PIPs following peripheral administration. Thus, we administered CWG-cPIP intracerebrally to investigate its effect on brain function in mouse models of CWG repeat diseases.

First, FITC-labeled CWG-cPIP (Supplemental Figure 2) was injected bilaterally into the mouse hippocampus, and its tissue distribution and retention for up to 7 days were assessed by histological analysis. FITC-labeled 83 μg/kg CWG-cPIP (1.5 nmol) was rapidly delivered to the cell nuclei of the hippocampus without any DDS and retained for at least 7 days. Moreover, we observed no cell death in the CWG-cPIP–injected hippocampus, as determined by cleaved caspase-3 immunoreactivity (Supplemental Figure 5).

The off-target effects of CWG-cPIP in vivo were investigated in the hippocampi 21 days after the treatment (83 μg/kg), and differentially expressed genes were detected only in 0.74% (Supplemental Figure 3B and Supplemental Data File 2). Among these genes, only *Inhbe* contained a (CTG)_16_ repeat, which is predominantly expressed in the liver (42).

**Figure 3:**
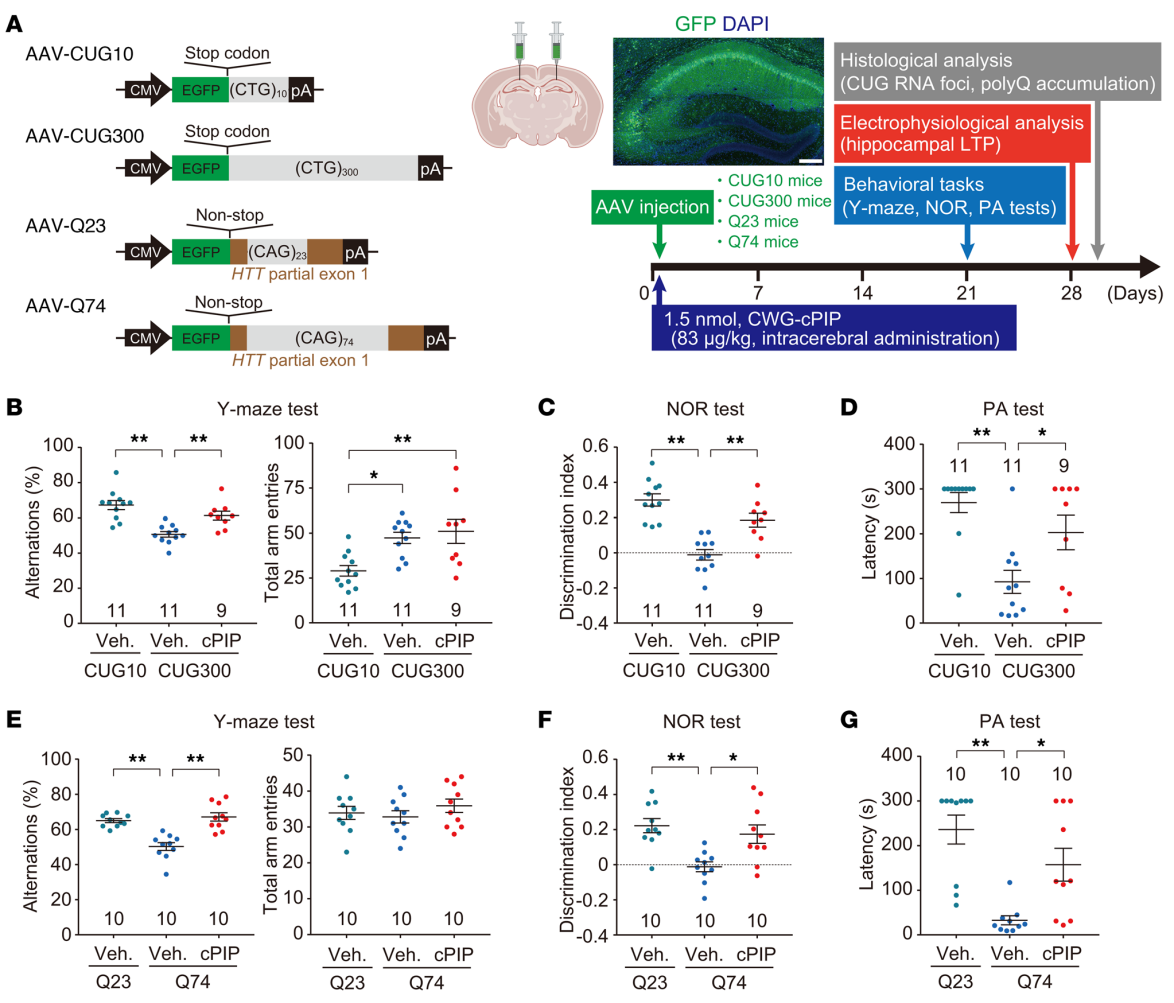
Amelioration of cognitive deficits observed in AAV-mediated CWG repeat–expressing mice by CWG-cPIP treatment. (**A**) Schematic representation of viral constructs used in in vivo experiments and experimental schedules and representative confocal image of GFP expression in the hippocampus of CUG10 mice. Scale bar: 200 μm. (**B** and **E**) Spontaneous alternation behaviors and locomotor activities in the Y-maze test. *P < 0.05 and **P < 0.01, by 1-way ANOVA with Bonferroni’s multiple-comparison test. CUG10 plus vehicle and CUG300 plus vehicle: *n* = 11 mice; CUG300 plus CWG-cPIP: *n* = 9 mice each (**B**); *n* = 10 mice each (**E**). (**C** and **F**) Discrimination indices for the NOR test sessions. *P < 0.05 and **P < 0.01, by 1-way ANOVA with Bonferroni’s multiple-comparison test. CUG10 plus vehicle and CUG300 plus vehicle: *n* = 11 mice; CUG300 plus CWG-cPIP: *n* = 9 mice each (**C**); *n* = 10 mice each (**F**). (**D** and **G**) Latency to enter the dark compartment in the PA test sessions. *P < 0.05 and **P < 0.01, by 1-way ANOVA with Bonferroni’s multiple-comparison test. CUG10 plus vehicle and CUG300 plus vehicle: *n* = 11 mice; CUG300 plus CWG-cPIP: *n* = 9 mice each (**D**); *n* = 10 mice each (**G**). Data represent the mean ± SEM. Statistical data are provided in Supplemental Data File 6.

To assess whether CWG-cPIP ameliorates brain dysfunction in CWG repeat diseases in vivo, we generated brain-specific and rapid-onset models through the following gene transfer into the bilateral CA1 region of the hippocampus using AAV serotype 9: insertion of (CTG)_10_ or (CTG)_300_ repeats into the 3′-UTR of *Egfp* mRNA (in the hippocampus of mice referred to herein as CUG10 and CUG300 mice), and EGFP-tagged (CAG)_23_ or (CAG)_74_ repeats within exon 1 of the *HTT* gene (in the hippocampus of mice referred to herein as Q23 and Q74 mice) (Figure 3A). CWG-cPIP did not affect the stability of recombinant AAV capsid proteins in vitro, nor did the AAV transduction efficiency when cotreated in HEK293 cells (Supplemental Figure 6). A mixture of CWG-cPIP (83 μg/kg) or vehicle and each AAV9 (1.0 × 10^13^ vector genomes/mL) was injected into the mouse hippocampus, and memory-related behaviors were evaluated in Y-maze, novel object recognition (NOR), and passive avoidance (PA) tests on days 21 to 27 after the injection. Hippocampal tissue was used for electrophysiology and immunohistochemistry on days 28 to 30 (Figure 3A).

In the Y-maze test, CUG300 and Q74 mice showed impaired memory-related behavior compared with CUG10 and Q23 mice. This was quantified by calculating the percentage of alternation behavior. The percentage of spontaneous alternation behavior significantly increased in CWG-cPIP–treated CUG300 and Q74 mice (Figure 3, B and E). CUG300 mice showed a characteristic behavior of dramatically increased locomotor activity, determined by the number of arm entries, and CWG-cPIP treatment did not improve hyperactivity (Figure 3B). In the NOR test, we observed no differences in the discrimination index using the same object for all mice during the training trials (Supplemental Figure 7A). After a 24-hour retention interval, CUG300 and Q74 mice had a significantly lower discrimination index for the novel object than did CUG10 and Q23 mice. The discrimination index for the novel object for CUG300 and Q74 mice treated with CWG-cPIP was significantly higher than that for the vehicle-treated mice (Figure 3, C and F). In the PA test, we observed no significant differences in latency to entering a dark room in the absence of a foot shock for all mice (Supplemental Figure 7B). However, latency to enter the dark compartment was markedly decreased 24 hours after foot shock for CUG300 and Q74 mice compared with CUG10 and Q23 mice. CWG-cPIP administration significantly restored the reduced latency time (Figure 3, D and G).

### CWG-cPIP ameliorates neuronal dysfunction in AAV-mediated, CWG repeat–expressing mice.

**Figure 4:**
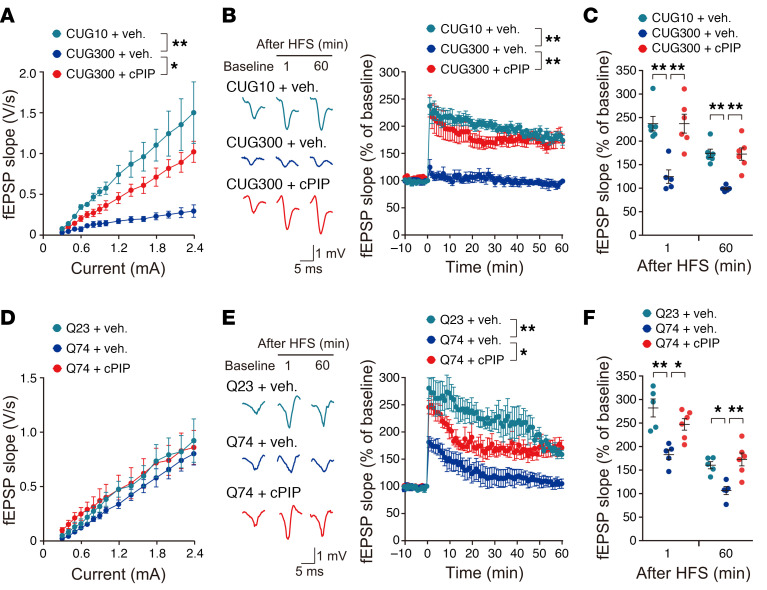
Mitigation of neuronal dysfunction observed in AAV-mediated CWG repeat–expressing mice following CWG-cPIP treatment. (**A** and **D**) Input-output curves generated from the field excitatory postsynaptic potential (fEPSP) slope in the hippocampal CA1 versus amplitude measured at increasing stimulus intensities. *P < 0.05 and **P < 0.01, by 2-way ANOVA with Bonferroni’s multiple-comparison test. CUG10 plus vehicle and CUG300 plus CWG-cPIP: *n* = 6 mice; CUG300 plus vehicle: *n* = 5 mice each (**A**); *n* = 5 mice each (**D**). (**B**, **C**, **E**, and **F**) Representative fEPSPs were recorded from the hippocampal CA1 region of mice (**B**, left; **E**, left). Representative fEPSP traces following HFS (**B**, right; **E**, right). (**C** and **F**) fEPSP slope changes following HFS at 1 or 60 minutes. **P < 0.01, by 2-way ANOVA with Bonferroni’s multiple-comparison test. CUG10 plus vehicle and CUG300 plus CWG-cPIP: *n* = 6 mice; CUG300 plus vehicle: *n* = 5 mice (**B** and **C**). *P < 0.05 and **P < 0.01, by 2-way ANOVA with Bonferroni’s multiple-comparison test. *n* = 5 mice each (**E** and **F**). Data represent the mean ± SEM. Statistical data are provided in Supplemental Data File 6.

We next assessed the electrophysiology of hippocampal long-term potentiation (LTP), which is critical for learning and memory. Interestingly, we found that basal synaptic transmission in input-output relationships was impaired in CUG300 compared with CUG10 mice (Figure 4A). In addition, we observed a dramatic reduction in high-frequency stimulation–induced (HFS-induced) LTP in CUG300 mice compared with that in CUG10 mice, and the reduced basal synaptic transmission and LTP in CUG300 mice were significantly restored following CWG-cPIP treatment (Figure 4, A–C). In Q74 mice, HFS-induced LTP was significantly impaired compared with that in Q23 mice without changes in basal synaptic transmission, and CWG-cPIP treatment significantly restored the reduction in synaptic plasticity observed in Q74 mice (Figure 4, D–F).

### CWG-cPIP inhibits nuclear CUG RNA foci and polyQ accumulation in AAV-mediated CWG repeat–expressing mice.

**Figure 5:**
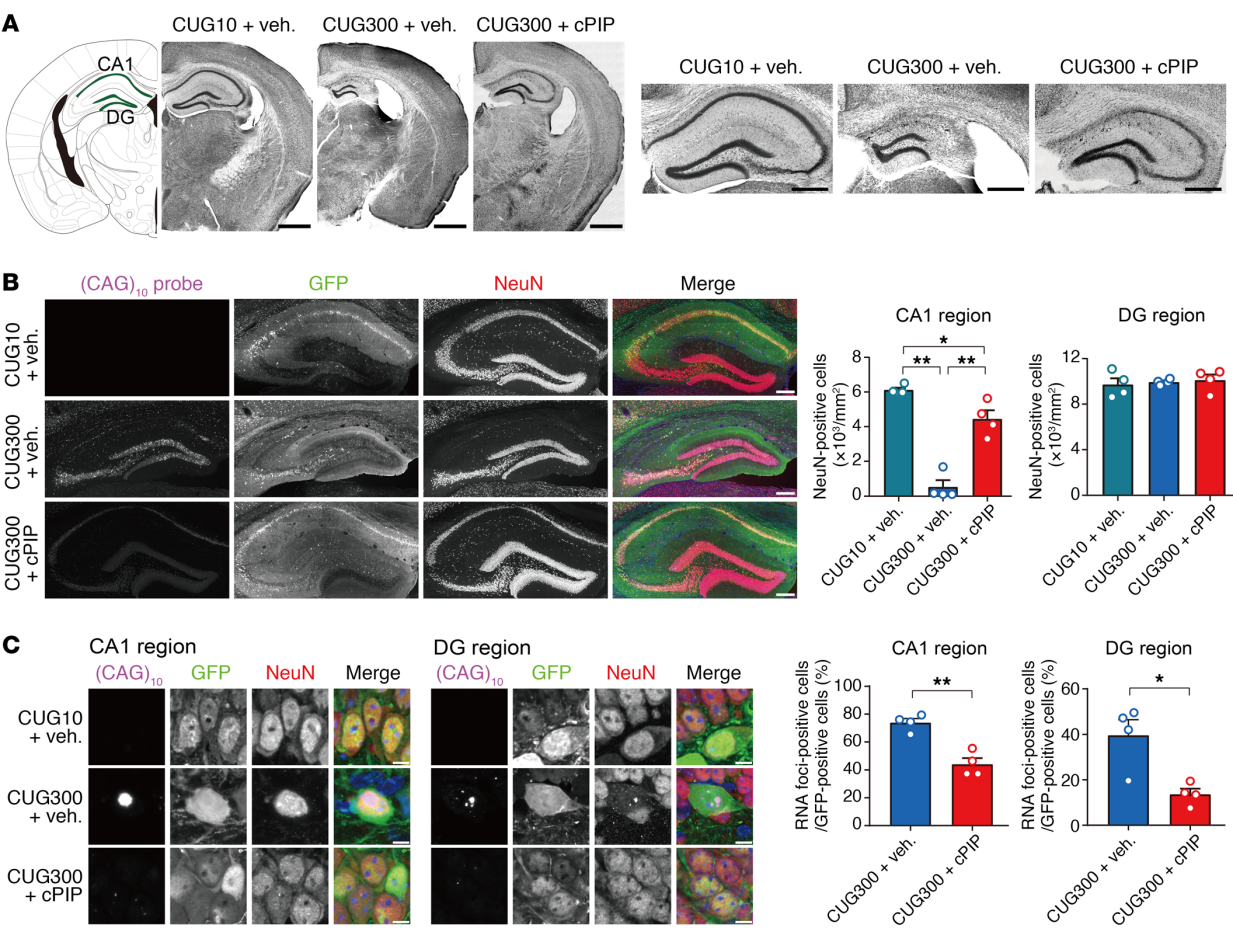
Inhibition of nuclear CUG RNA foci seen in CUG300 mice by CWG-cPIP treatment. (**A**) Representative confocal images of Nissl-stained sections. Scale bars: 1 mm (left) and 500 μm (right). (**B**) Representative confocal images of CUG-RNA (magenta), GFP (green), and NeuN (red) in the hippocampus and quantification of NeuN-positive cells in CA1 and DG regions. *P < 0.05 and **P < 0.01, by 1-way ANOVA with Bonferroni’s multiple-comparison test. *n* = 4 mice each, averaged from 3 independent replicates (*n* = 3 slices) per mouse. Scale bars: 200 μm. (**C**) Representative confocal images of CUG-RNA foci in the hippocampal CA1 and DG regions and their quantification. *P < 0.05 and **P < 0.01, by 2-sided, unpaired Student’s *t* test. *n* = 4 mice each, averaged from 3 independent replicates (*n* = 3 slices) per mouse. Scale bars: 5 μm. Data represent the mean ± SEM. Statistical data are provided in Supplemental Data File 6.

First, the pathological changes in the brains of AAV-mediated CWG repeat–expressing mice were evaluated using Nissl staining. Nissl staining revealed obvious hippocampal atrophy in CUG300 mice compared with that in CUG10 mice (Figure 5A), whereas no significant changes were observed between Q23 and Q74 mice (Supplemental Figure 8A). In the immunohistochemical study, we assessed the immunoreactivity of NeuN, a neuronal marker. The number of NeuN-positive cells was significantly reduced in the hippocampal CA1 region of CUG300 mice compared with CUG10 mice (Figure 5B). In contrast, we observed no significant difference in the number of NeuN-positive cells in the hippocampal dentate gyrus (DG) region between CUG10 and CUG300 mice. Importantly, CWG-cPIP treatment significantly improved the reduced number of NeuN-positive cells in the hippocampal CA1 region of the CUG300 mice (Figure 5B). Next, we evaluated the number of CUG RNA foci–positive cells relative to GFP-positive cells by FISH using a Cy5-labeled (CAG)_10_ probe. Consistent with the cell culture experiments, we observed CUG RNA foci in the hippocampal CA1 and DG regions of CUG300 mice but not in those of CUG10 mice. In addition, CWG-cPIP treatment significantly decreased the number of CUG RNA foci in CUG300 mice (Figure 5C).

Unlike the tissue damage observed in CUG300 mice, we found no significant changes in the number of NeuN-positive cells in the hippocampal CA1 and CA3 regions of Q74 mice compared with those in Q23 mice (Supplemental Figure 8B). Immunohistochemical analysis of polyQ inclusions in GFP-positive cells revealed several polyQ inclusions in the hippocampal CA1 and CA3 regions of Q74 but not Q23 mice. Treatment with CWG-cPIP significantly reduced the number of polyQ inclusions in Q74 mice (Supplemental Figure 8C).

### CWG-cPIP restores dysregulation of alternative splicing and gene expression changes in CUG300 mice.

**Figure 6:**
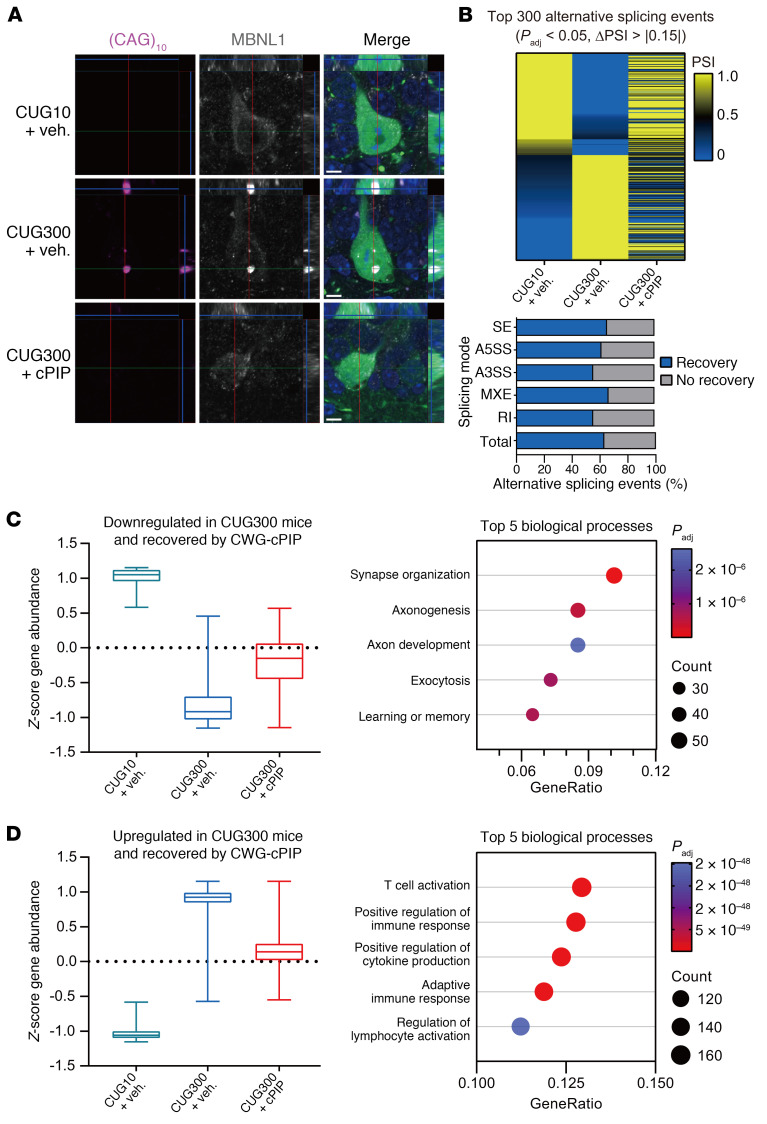
Restoration of splicing defects and gene expression changes seen in CUG300 mice following CWG-cPIP treatment. (**A**) Representative confocal images of CUG-RNA foci (magenta), MBNL1 (white), and GFP (green) in the hippocampus. Scale bars: 5 μm. (**B**) Heatmap of the top 300 (sorted by PSI of CUG10 mice) differential alternative splicing events and stacked bar chart showing the percentage of improvement over the total and each splicing mode in CUG300 mice after treatment with CWG-cPIP. *n* = 1 mouse each. Source data are provided in Supplemental Data File 3. (**C** and **D**) *Z* score–converted |expression levels of genes downregulated (**C**) and upregulated (**D**) in CUG300 mice and restored by CWG-cPIP treatment. Lines in the middle of the boxes indicate the median, and the top and bottom of the whiskers indicate the maximum and minimum values, respectively. Graphs on the right show the top 5 enriched gene ontology biological processes. *n* = 3 mice each. Source data are provided in Supplemental Data File 4. *P*_adj_, adjusted *P* value.

In the brains of patients with DM1, mutant *DMPK* RNA accumulates extensively as nuclear RNA foci, sequestering RNA-binding proteins such as MBNL proteins and affecting their function, which leads to splicing defects in a variety of pre-mRNAs and misexpression of different protein isoforms (43, 44). To assess the effect of CWG-cPIP on MBNL1 sequestration, we examined CUG RNA foci formation and nuclear MBNL1 localization in the hippocampus of CUG300 mice in the presence or absence of CWG-cPIP. CUG10 mice showed diffuse localization of MBNL1 throughout the cytoplasm and nucleus, whereas in CUG300 mice, MBNL1 was sequestered in the nuclear CUG RNA foci. Treatment with CWG-cPIP resulted in the redistribution of MBNL1 along with the elimination of CUG RNA foci (Figure 6A).

We further assessed whether treatment with CWG-cPIP could restore the dysregulation of gene alternative splicing and gene expression changes observed in CUG300 mice. To investigate these changes prior to neuronal loss, we performed RNA-Seq analysis of the hippocampi of mice 10 days after gene transfer via AAV9 and focused on the top 300 differential alternative splicing events (adjusted *P* < 0.05, percent-spliced-in [PSI] difference [ΔPSI]between CUG10 and CUG300 groups > |0.15|) (Figure 6B and Supplemental Data File 3). The top 300 events were classified into the following 5 differential splicing modes: 202 skipping exon (SE), 36 alternative 5′ splice site (A5SS), 47 alternative 3′ splice site (A3SS), 6 mutually exclusive exons (MXEs), and 9 retention introns (RIs). Compared with vehicle treatment, CWG-cPIP treatment led to the recovery of more than half of the events in all splicing modes and recovered, overall, 63% of the top 300 differential alternative splicing events between CUG10 and CUG300 mice (Figure 6B). Gene expression analysis revealed 2,000 differentially expressed genes between all groups (Supplemental Data File 4). Most genes were clustered into 2 groups according to the direction of change in expression levels: 551 genes were downregulated in CUG300 mice and recovered by CWG-cPIP treatment (Figure 6C), and 1,349 genes were upregulated in CUG300 mice and recovered by CWG-cPIP treatment (Figure 6D). Gene enrichment analysis further suggested that, while downregulated genes in CUG300 mice contribute to synaptic and cognitive functions, upregulated genes participate in the immune response (Figure 6, C and D).

### CWG-cPIP improves behavioral and pathological impairments in a genetic mouse model of HD.

Finally, we assessed the potential of i.c.v. administration of CWG-cPIP to alleviate neurological symptoms and pathology in R6/2 mice, a commonly used genetic mouse model of HD to evaluate new drugs against this disease (45, 46). Prior to this, we validated the nuclear translocation, cell damage, and off-target effects of i.c.v. administered CWG-cPIP. FITC-labeled CWG-cPIP (664 μg/kg) was bilaterally injected into the lateral ventricles of WT mice, and its tissue distribution in the striatum, the region most affected by HD pathology, was examined (11). We observed the signal in the cell nuclei of the striatum, and it remained detectable for at least 7 days. Furthermore, we observed no cell damage based on cleaved caspase-3 immunoreactivity (Supplemental Figure 9). In addition, RNA-Seq analysis revealed no differentially expressed genes in the striatum 21 days after administration of CWG-cPIP (Supplemental Figure 3C and Supplemental Data File 5).

**Figure 7:**
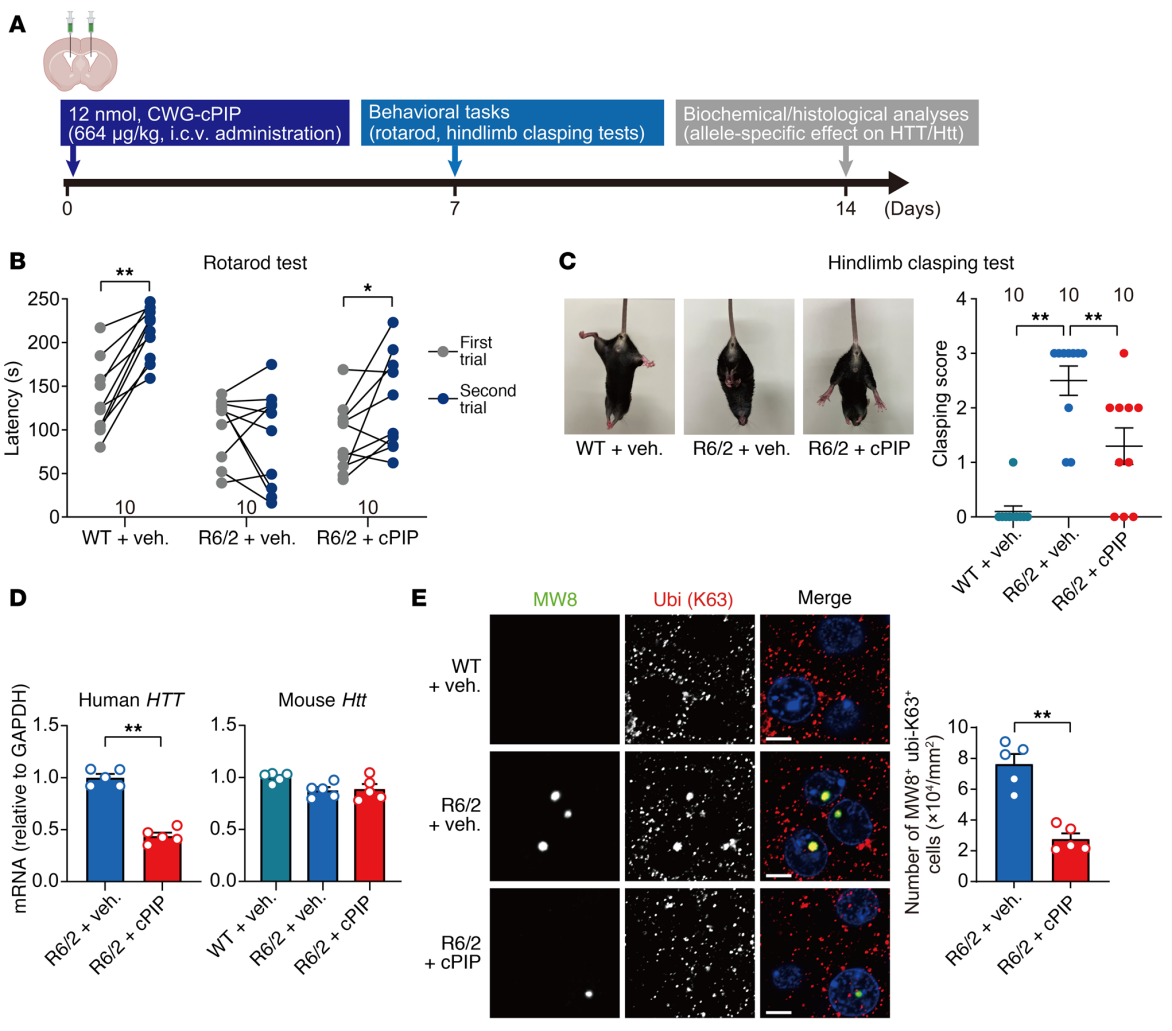
Improvement of neurological symptoms and pathology seen in R6/2 mice following CWG-cPIP treatment. (**A**) Experimental diagram of i.c.v. injection of CWG-cPIP into R6/2 mice and the corresponding schedule. (**B**) Latency to fall for each trial in the rotarod test. *P < 0.05 and **P < 0.01, by 2-sided, paired Student’s *t* test. *n* = 10 mice each. (**C**) Representative images of hind limb clasping and quantification of the clasping score in the hind limb clasping test. **P < 0.01, by 1-way ANOVA with Bonferroni’s multiple-comparison test. *n* = 10 mice each. (**D**) Quantification of endogenous mouse *Htt* and human *HTT* transgene mRNA levels in the striatum. **P < 0.01, by 2-sided, unpaired Student’s *t* test (left); multiple comparisons were performed by 1-way ANOVA with Bonferroni’s multiple-comparison test (right). *n* = 5 mice each. (**E**) Representative confocal images of MW8 and K63-ubiquitin in the striatum and quantification. Scale bars: 5 μm. **P < 0.01, by 2-sided, paired Student’s *t* test. *n* = 5 mice each, averaged from 12 replicates per 100 μm^2^ area each in 3 slices per mouse. Data represent the mean ± SEM. Statistical data are provided in Supplemental Data File 6.

Next, R6/2 mice were administered CWG-cPIP (664 μg/kg) i.c.v., and rotarod and hind limb clasping tests were conducted 1 week later. Striatal tissue was used for biochemical and immunohistochemical analysis after another week (Figure 7A). In the behavioral tests, R6/2 mice were impaired in motor skill learning over trials and showed severe clasping phenotypes in the hind limbs, and these neurological symptoms in R6/2 mice were significantly improved by CWG-cPIP (Figure 7, B and C). We then examined the effects of CWG-cPIP on the production of the pathogenic human *HTT* transgene and endogenous mouse *Htt* in R6/2 mice using reverse transcription quantitative PCR (RT-qPCR) analysis. CWG-cPIP had inhibitory effects on *HTT* transgene transcript expression but had no effect on endogenous *Htt* transcript levels (Figure 7D). We also analyzed the effects of CWG-cPIP by histochemistry. MW8-positive HTT aggregates were found to accumulate in the striatum of R6/2 mice, colocalizing with K63-specific ubiquitin, which promoted HTT aggregation (47). CWG-cPIP substantially reduced the K63-ubiquitinated HTT aggregates (Figure 7E).

## Discussion

In this study, we identified CWG-cPIP as a candidate compound for CWG repeat diseases. We demonstrate that CWG-cPIP exhibited high binding affinity for CWG DNA and was preferentially active on repeat-expanded DNA. CWG-cPIP markedly suppressed the production of pathogenic CUG RNA foci and polyQ in neurons, ameliorating neuronal dysfunction and cognitive impairment in AAV-mediated CWG repeat–expressing mice. Furthermore, the production of pathogenic *HTT* mRNA and protein was attenuated in R6/2 mice, a genetic model of HD. In addition, CWG-cPIP exhibits many useful properties, including negligible toxicity, easy nuclear translocation without the need for DDS, and few off-target effects and may be used as a therapeutic agent for CWG repeat diseases.

Although CWG repeat diseases have been known for several decades, their pathological mechanisms are unclear, and no standard treatment has been established to date. Recent studies have identified the pathological hypotheses of RNA toxicity, polyQ toxicity, and RAN translation in the manifestation of CWG repeat diseases (3, 5). On the basis of these findings, several therapeutic approaches have been developed, including pharmacological compounds, stem cell–based therapies, and gene therapies (48, 49). CRISPR/Cas9 system–mediated genome editing has been shown to eliminate the expanded CWG repeat DNA in DM1 (50, 51) and HD (52–55). However, when DNA breaks occur near repeat sequences, the repair machinery is activated, which leads to expansion growth and may cause further mutation of the repeats (56). Alternatively, CRISPR-based technologies capable of perturbing CWG repeat RNA have been extensively evaluated as potential therapeutics (57–59). However, because Cas proteins are bacterial in origin, they can be recognized as foreign by the immune system, and long-term expression may trigger an autoimmune response (60).

Elimination of the pathogenic repeat RNAs using ASOs may avoid these issues (26). Preclinical and clinical trials are currently underway for ASO therapeutics that target pathogenic RNAs in CWG repeat diseases (27). Clinical trials using ASOs for HD are the most advanced in terms of restoring brain function, however, a series of recent ASO trials have been terminated. In 2021, Roche announced the early completion of a phase III trial of its ASO drug for HD, tominelsen (28). Wave Life Sciences also reported that the 2 ASO candidates for HD did not slow the disease progression in phase I/II clinical trials (29). Several factors may have contributed to these failures. For example, ASOs may cause problems by suppressing the production of normal and mutant forms of HTT (61), or ASOs may not even reach the appropriate areas of the brain (62). Moreover, because ASOs are easily degraded by nucleases, suitable chemical modifications or DDSs are required for their therapeutic applications.

Here, we demonstrated that CWG-cPIP is a potential therapeutic agent for solving the clinical challenges associated with CRISPR and ASO technologies owing to its DNA sequence specificity, repeat length preference, nuclear localization, and low toxicity. Furthermore, RNA-Seq analysis using external standards revealed that CWG-cPIP had no significant effect on global transcription. Notably, PIPs are completely resistant to nucleases (63) and can be delivered into tissues without a DDS. Although we demonstrated the efficacy of brain parenchymal and i.c.v. administration of CWG-cPIP in mouse models, the efficacy of intrathecal administration is yet to be established. Bypassing the blood-brain barrier, intrathecal administration enables the direct delivery of therapeutic agents into the cerebrospinal fluid, which circulates within the brain to ensure delivery to the brain regions needing therapy. Although more invasive, this approach allows the administration of lower doses than those required for systemic deliveries, minimizing the risk of toxicity; the i.v. dose must be approximately 100 times higher than the i.c.v. dose (64). The most promising results for ASO-based therapy have been obtained through direct ASO administration via intrathecal delivery of agents such as the FDA-approved nusinersen for spinal muscular atrophy (65). The versatility of PIPs, attributed to their ease of synthesis and modification, has led to the establishment of venture companies in the United States (GeneLab, GeneSoft, and Design Therapeutics), the United Kingdom (Spirogen), and Japan (Gentier Biosystems, Regugene) for the medical application of PIPs. Notably, DT-216, a PIP that facilitates the transcription of repressive GAA repeats to enhance frataxin expression in Friedreich’s ataxia, is currently undergoing phase I clinical trials (NCT05285540) (66). CWG-cPIP can serve as a lead compound for the synthesis of an ideal therapeutic for efficient and specific elimination of pathogenic repetitive transcripts through collaboration between industry and academia.

HD has long been recognized as a cause of neuronal death, mainly resulting in striatal atrophy and degeneration of the medium spiny neurons (67). The hippocampus is also a pathological region in HD, and impaired cognitive function related to the hippocampus is believed to contribute to disease onset (68, 69). Although DM1 research has predominantly focused on progressive muscle weakness and myotonia, interest in the neurological aspects of DM1 has grown in recent years because of their impact on the quality of life of DM1 patients (70). Brain imaging in patients with DM1 has revealed white matter abnormalities, extensive gray matter atrophy, and hypometabolism in the frontal lobe (71). In addition, executive, memory and visuospatial deficits are associated with a decrease in total brain volume (72). As in skeletal muscles, nuclear CUG RNA foci colocalized with MBNL1 and MBNL2 have been detected in the brains of patients with DM1, and loss of function of MBNL proteins due to their sequestration is a key factor in DM1 neuropathology. *Mbnl1*- and *Mbnl2*-KO mice recapitulate some DM1 neuropathological phenotypes, including dysregulated RNA processing and spatial learning deficits (73, 74).

We found that endogenous MBNL1 was localized in the nucleus and cytoplasm in CUG10 mouse brains without CUG RNA foci, however, in CUG300 mouse brains, characteristic nuclear RNA foci had formed, which sequestered MBNL1 (Figure 6A). In addition, the hippocampi of CUG300 mice showed alternative splicing defects compared with those of CUG10 mice (Figure 6B). Based on these results, the CUG300 mouse could be considered a murine model that reflects the brain dysfunction of human DM1. Although we could not address the direct involvement of splicing abnormalities and neurodegeneration, misspliced candidates, such as *GRIN1*, *MAPT*, and *APP*, have been reported in the DM1 brain (21). Exons 2 and 10 of *MAPT* are misspliced in DM1 brains, resulting in the preferential accumulation of the 0N3R isoform (75). Missplicing of *MAPT* leads to tauopathy, with tau aggregation and neurofibrillary tangles (76). Furthermore, multiple protein deposits, including granulovacuolar degeneration and skein-like ubiquitin-positive inclusions, have been observed in DM1 brains (77). Gene ontology enrichment analysis revealed that synaptic and cognitive functions were dysregulated in CUG300 mice (Figure 6C), consistent with the results of behavioral and electrophysiological analyses (Figures 3 and 4). Interestingly, the dysregulated genes in CUG300 mice were also enriched in the immune system (Figure 6D). Consistent with this, upregulated genes in the lens epithelia of patients with DM1 were enriched in the innate immune response, and the changes in the immune response system have been suggested to correlate with disease severity (78, 79). Immune dysfunction, such as T cell activation and cytokine production, is also a key event in neurodegenerative diseases, including Alzheimer’s disease and Parkinson’s disease (80). The relationship between splicing abnormalities and the immune system with neurodegeneration in the DM1 brain should be further studied.

We used AAV-treated mice as a model of CWG repeat diseases, as they are suitable as a short-term model for in vivo drug efficacy evaluation. An AAV-induced model manufactured to express Q97-GFP in the adult rat brain has been shown to rapidly form nuclear polyQ aggregates in neurons (81), which is similar to what we observed in Q74 mice (Supplemental Figure 8). Moreover, we established a new AAV-induced CTG repeat–expressing model (CUG300 mice), in which a (CTG)_300_ repeat was inserted into the 3′-UTR of *Egfp* mRNA. Notably, histological analysis revealed that CUG300 mice had much greater neuronal damage than did Q74 mice (Figure 5). The histological results were consistent with the significantly reduced basal synaptic transmission of input-output relationships in CUG300 mice compared with those in CUG10 mice (Figure 4). Furthermore, treatment with CWG-cPIP restored the splicing defects, synaptic dysfunction, and memory impairment observed in CUG300 mice. To further explore the clinical potential of drug effects, we investigated CWG-cPIP effects using R6/2 mice as a more clinically relevant model (46). Notably, we found that CWG-cPIP remarkably inhibited the production of pathogenic HTT mRNA and protein as early as 2 weeks after administration (Figure 7). However, since human DM1 and HD slowly progress through neurodegeneration over the decades, it would be difficult to detect human-like changes in disease progression through overexpression systems using AAV-infected mice and transgenic mice. Other limitations of this study include the lack of long-term evaluation of CWG-cPIP. ASO and CRISPR/Cas13 have been evaluated in HD animal models over a wide range of treatment durations from 1 week to several months and have been reported to improve neurological symptoms by eliminating mutant *HTT* mRNA (59, 82, 83) but have not yet been clinically successful. Further studies are thus needed to address whether CWG-cPIP is effective against more pathological conditions similar to those seen in patients, including endogenous pathogenic repeat DNA, using induced pluripotent stem cells and CWG repeat–knockin models with more slowly progressive symptoms such as zQ175 mice (46) and those harboring DMPK with (CTG)_480_ repeats (84).

In conclusion, we demonstrated that CWG-cPIP is a safe therapeutic candidate for CWG repeat diseases that effectively suppresses pathogenic CUG RNA foci and polyQ at the transcriptional level. CWG-cPIP exhibits high binding capacity for the CWG repeat DNA sequence, and its administration substantially restored the molecular, physiological, and behavioral impairment associated with CWG repeat diseases. The long-term efficacy and efficiency of intrathecal administration of CWG-cPIP need to be investigated in the future using large mammalian models.

## Methods

### Study design.

The present study aimed to assess the effects of CWG-cPIP on CWG triplet repeat diseases. To this end, we designed in cellulo and in vivo models based on disease pathology and conducted in vitro studies to assess the affinity and specificity of CWG-cPIP binding to targeted DNA sequences, including in the Neuro-2a cell line, mouse primary cultured neurons, and patient-derived fibroblasts and iNeurons.

WT mice (C57BL/6J and ICR; Japan SLC) were housed under climate-controlled conditions on a 12-hour light/12-hour dark cycle and were provided standard food and water ad libitum. Male R6/2 mice were purchased from The Jackson Laboratory (stock no. 006494). To maintain this strain, R6/2 Tg sperm were fertilized in vitro using C57BL/6J eggs and implanted into ICR mice. The pups were regularly genotyped for the human HTT exon 1 transgene and the length of the CAG repeat. The average CAG repeat length in the R6/2 mice used in this study was 124 (maximum, 132; minimum, 121). WT littermates were used as controls and were housed in mixed-genotype and single-sex cages under the conditions described above. All experiments using animals and human samples followed the institutional guidelines and were approved by the institutional committee. Because mice with a WT C57BL/6 background show significant differences based on sex in object and spatial recognition (85), AAV models were consistently developed using male mice. However, as there was no sex difference in the decline pattern of motor performance or the amount of mutant HTT in the brain of R6/2 mice (86), both sexes were used for this study. R6/2 mice aged 7–9 weeks and their WT counterparts were used for i.c.v. administration of CWG-cPIP.

Animal models were established by expressing disease-causing repeat DNA using an AAV system in addition to a genetically engineered HD mouse model. Animals were randomly assigned to treatment groups, and investigators were blinded to the group allocation during behavioral analysis. The sample size was empirically determined on the basis of pilot and previous studies with the relevant fields reported in the literature. No data were excluded as outliers. The statistical data are summarized in Supplemental Data File 6. Detailed methods are described in the Supplemental Methods.

### Statistics.

All values are expressed as the mean ± SEM unless otherwise indicated. Statistical significance of differences among groups was tested by 1-way or 2-way ANOVA with post hoc Bonferroni’s multiple-comparison test. Comparisons between 2 experimental groups were performed using a 2-sided, unpaired Student’s *t* test. Statistical significance was set at a *P* value of less than 0.05. All statistical analyses were performed using GraphPad Prism 7 (GraphPad Software).

### Study approval.

Animal studies were conducted in accordance with Kumamoto University institutional guidelines. Ethics approval was obtained from the IACUC of the Kumamoto University Environmental and Safety Committee (approval no. A2020-022). Human fibroblasts were obtained from the NIGMS Human Genetic Cell Repository at the Coriell Institute for Medical Research with approval for use given by the research ethics committee of Kumamoto University (approval no. 1842).

### Data availability.

Values for all data points in graphs are reported in the Supplemental Supporting Data Values file. All statistical data are shown in Supplemental Data File 6. The raw data from the RNA-Seq analysis are available in the NCBI’s Gene Expression Omnibus (GEO) database (GEO GSE210839). Additional data related to this work may be requested from the corresponding author upon reasonable request.

## Author contributions

HS and NS conceived the study. SI, KM, YY, KK, SA, KH, SU, and YH performed the experiments. TB, KA, and HS provided the resources. TB, MU, HS, and NS supervised the study. SI, KM, and NS wrote the original draft, and all authors reviewed and edited the manuscript. The order of the co–first authors’ names was decided on the basis of the level of involvement of SI from the conception of the study.

## Supplementary Material

Supplemental data

Supplemental data set 1

Supplemental data set 2

Supplemental data set 3

Supplemental data set 4

Supplemental data set 5

Supplemental data set 6

Supporting data values

## Version 1 09/14/2023

In-Press Preview

## Version 2 11/15/2023

Electronic publication
