# Supplementary material for: A cyclic pyrrole-imidazole polyamide reduces pathogenic RNA in CAG/CTG triplet repeat neurological disease models
Source: J Clin Invest. 2023 Nov 15;133(22):e164792. doi: 10.1172/JCI164792 (PMC10645379; doi:10.1172/JCI164792)
Supplement: Supplemental data [file jci-133-164792-s139.pdf]

Supplemental materials:

Methods

Synthesis of PIP compounds

The reagents and solvents were purchased from standard suppliers and used without further purification. HPLC analysis of the compounds was performed on a Jasco Engineering PU-2089 plus series system using a COSMOSIL 150×4.6 mm 5C_{18}-MS-II Packed Column (Nacalai Tesque, Inc.) in 0.1% trifluoroacetic acid in water with acetonitrile as the eluent at a flow rate of 1.0 mL/min and a linear gradient elution of 0–100% acetonitrile in 40 min with detection at 254 nm. The collected fractions were analyzed using MALDI-TOF MS microflex-KS II (Bruker).

CWG-cPIP and CWG-hPIP were synthesized as reported previously (37) and purified using the CombiFlash Rf RFJ model with RediSep Rf 4.3 g C18 reverse-phase column (Teledyne Isco, Inc.).

To obtain FITC-labeled CWG-cPIP, Cbz-protected cPIP, cyclo-(-ImPyβImPy-(*R*)$^{\alpha\text{-}NH2}$γ-ImPyβImPy-(*R*)$^{\alpha\text{-}NHCbz}$γ-), was synthesized following procedures similar to those described previously (36). The crude sample was dissolved in *N, N*-dimethylformamide (DMF), and Fmoc-mini-PEG™ (1.5 equiv.; Peptides International, Inc.), pentafluorophenyl diphenylphosphinate (1.5 equiv.) and diisopropylethylamine (DIEA, 3 equiv.) were added to it, and the mixture was then stirred for 4 h at room temperature. The mixture was dropped into Et_2O and subjected to centrifugation, following which Et_2O was removed and the pellet was dried in vacuo. The Fmoc protecting group was removed by 20% piperidine/DMF treatment for 30 min at room temperature. The mixture was precipitated in Et_2O and the

resulting powder was dried in vacuo. The pellet was dissolved in DMF with fluorescein 5-isothiocyanate (2 equiv.) and DIEA (6 equiv.), and the mixture was stirred for 2 h at room temperature. After the workup, the Cbz protecting group was removed using trifluoromethanesulfonic acid/trifluoroacetic acid (1:10) treatment for 4 min at room temperature. Workup with Et_2O gave a crude powder of cyclo-(-ImPyβImPy-$(R)^{\alpha\text{-NH2}}$γ-ImPyβImPy-$(R)^{\alpha\text{-NH-miniPEG-FITC}}$γ-). After purification, 5.4 mg of the sample was obtained (2.9 μmol, 9% yield for 18 steps). Analytical HPLC: t_R=17.7 min. MALDI-TOF MS: *m/z* calcd. for $C_{85}H_{93}N_{28}O_{20}S^+$ $[M+H]^+$ 1857.68, found; 1857.74. The HPLC and MALDI-TOF MS spectra of FITC-labeled CWG-cPIP are shown in Supplementary Figure 2.

Structural model of CWG-cPIP binding to DNA

Molecular modeling studies were performed with Discovery Studio (BIOVIA) using the charmm27 force field. The initial PIP structure was built based on previous crystal structures (PBD ID: 3I5L) and manually inserted into the minor groove of the B-DNA sequence 5′-GCAGCAGCAGC-3′/3′-CGTCGTCGTCG-5′ constructed using the builder module. The complex was solvated in cubic water with 50 mM NaCl and pre-minimized to maintain the interaction distance of hydrogen bonds between the polyamide moiety and DNA base pairs. Then, A and T in the sequence were replaced with each DNA base to obtain the B-DNA sequence (5′-GCNGCNGCNGC-3′/ 3′-CGNCGNCGNCG-5′, N = A, T, G, C). The entire structure was finally minimized to the stage where the root-mean-square was less than 0.001 kcal/mol·Å using the conjugate gradient algorithm with no constraint.

Melting temperature T_m assay

DNA and RNA oligomers were purchased from Fasmac and Hokkaido System Science, respectively:1) d(CAG/CTG) (5′-CGAGCAGCACG-3′/5′-CGTGCTGCTCG-3′); 2) d(CGG/CCG) (5′-CGGGCGGCGCG-3′/5′-CGCGCCGCCCG-3′); 3) AT rich (5′-CGATTATTACG-3′/5′-CGTAATAATCG-3′) 4) GC rich (5′-CGGCGCCGCCG-3′/5′-CGGCGGCGCCG-3′); 5) 5′-d$(CAG)_{10}$ repeat-3′; 6) 5′-d$(CTG)_{10}$ repeat-3′; 7) 5′-d$(CGG)_{10}$ repeat-3′; 8) 5′-d$(CCG)_{10}$ repeat-3′; 9) 5′-r$(CAG)_{10}$ repeat-3′; 10) 5′-r$(CUG)_{10}$ repeat-3′. The analytical buffer used for the T_m assay was an aqueous solution of NaCl (2.5 mM) and Tris-HCl (10 mM) at pH 7.5 containing 0.375% DMSO. The concentrations of double-stranded DNA, mismatched hairpin DNA, and mismatched hairpin RNA were 2.5 μM. The concentration of polyamides was 3.75 μM (1.5 equiv.). Before the analysis, the samples were annealed from 95°C to 20°C at a rate of 1.0°C/min, and the absorbance at 260 nm was recorded from 20°C to 95°C at a rate of 1.0°C/min using a spectrophotometer (V-750; JASCO, Inc.) with a thermocontrolled cell changer (PAC-743R; JASCO, Inc.) and a thermal circulator (CTU-100; JASCO, Inc.). The T_m values shown in Figure 1B are the averages of all data. The calculated T_m and ΔT_m values are presented in Supplementary Table 1.

Plasmid constructs

For transcription arrest assay, a random sequence of 189 base pairs with $(CTG)_{10}$ repeats at the N-terminus or a $(CTG)_{73}$ repeat sequence was subcloned into pcDNA3.1(+), termed pT7$(CTG)_{10}$ or pT7$(CTG)_{73}$, respectively. The $(CTG)_{73}$ sequence was obtained from plasmid pAAV-CTG700x (#63087; Addgene). Random DNA sequence was synthesized

commercially by Eurofins Genomics. To evaluate the production of CWG repeat RNAs in cells, we generated a dual promoter vector pFC-EF1-MCS-pA-PGK-EGFP using PhiC31 vector (FC551A-1; System Biosciences, LLC) as a backbone. For HaloTag-CTG repeat mRNA expression plasmid, a subcloned fragment with $(CTG)_{10}$, $(CTG)_{180}$, or $(CTG)_{700}$ repeat sequence in the 3′-UTR of HaloTag was inserted into the MCS of pFC-EF1-MCS-pA-PGK-EGFP vector, termed CUG10, CUG180 or CUG700, respectively. These CTG repeat sequences were obtained from plasmid pAAV-CTG700x (#63087; Addgene). For HaloTag-CAG repeat mRNA expression plasmid, a subcloned fragment of $(CAG)_{23}$ or $(CAG)_{74}$ repeat sequence within exon 1 of the *HTT* gene was inserted into the MCS of pFC-EF1-MCS-pA-PGK-EGFP vector. These CAG repeat sequences were obtained from plasmid pEGFP-Q23 and pEGFP-Q74 (#40261 and #40262, respectively; Addgene). For EGFP-CTG repeat mRNA expression plasmid, a subcloned fragment with $(CTG)_{10}$ or $(CTG)_{700}$ repeat sequences in the 3′-UTR of *Egfp* were inserted into the MCS of pCAG-Neo vector (Wako Pure Chemical). For EGFP-CTG repeat mRNA expression AAV vector plasmid, a fragment with $(CTG)_{10}$ or $(CTG)_{300}$ repeat sequence in the 3′-UTR of *Egfp* was subcloned into plasmid pAAV-CTG700x (#63087; Addgene), termed pAAV-CUG10, pAAV-CUG300, respectively. For EGFP-CAG repeat (EGFP-polyQ) expression AAV vector plasmid, a fragment of *Egfp* with $(CAG)_{23}$ or $(CAG)_{74}$ repeat sequence at the C-terminus was subcloned into plasmid pAAV-CTG700 (#63087; Addgene), termed pAAV-Q23 or pAAV-Q74, respectively.

Transcription arrest assay

The $pT7(CTG)_{10}$ and $pT7(CTG)_{73}$ plasmids were linearized using EcoRI restriction enzyme and purified using the Wizard SV Gel and PCR Clean-Up System (Promega). Transcription arrest assays were performed using HiScribe T7 high-yield RNA synthesis kit (New England Biolabs) with 0.5% DMSO (vehicle) or CWG-cPIP (1.25, 2.5, or 3.75 μM), and 200 ng of the linearized plasmid was obtained, which produces a 321-base RNA under the T7 promoter. After transcription for 10 min at 37°C, DNase I was added according to the manufacturer's instructions. Transcription products were analyzed by urea-denaturing polyacrylamide gel electrophoresis on 7% gels containing 7 M urea at 200 V for 120 min. Before loading, samples were heated for 4 min at 90°C with RNA Loading Dye (New England Biolabs), and then immediately cooled on ice for a few minutes. After the electrophoresis, the gels were stained with SYBR Gold (Invitrogen) for 20 min and visualized using Typhoon Trio equipment (GE Healthcare).

Cell culture

Cell cultures were established according to previously described methods (87). The Neuro-2a mouse neuroblastoma cell line CCL-131 was authenticated by the provider using short tandem repeat profiling (American Type Culture Collection) and was grown in DMEM (Sigma-Aldrich) supplemented with 10% FBS (Gibco) and 1× penicillin/streptomycin (Gibco) in a 5% CO_2 incubator at 37°C. Transfection was performed using the Lipofectamine 2000 Transfection Reagent (Invitrogen) according to the manufacturer's protocol. For the primary culture of neurons, cortical tissue was dissected and dispersed from the mice on embryonic day 18. Cells were seeded on coverslips coated with poly-L-lysine in MEM

(Thermo Fisher Scientific) supplemented with 10% FBS, 0.6% glucose (Wako Pure Chemical), and 1 mM pyruvate (Sigma-Aldrich). After cell attachment, the cells were cultured in Neuron Culture Medium (Wako Pure Chemical) in a 5% CO_2 incubator at 37°C. Cultured neurons were transfected with plasmids using an electroporator (NEPA21; Nepa Gene) on day 0 in vitro (DIV0), and subjected to biochemical experiments on DIV14. Human fibroblasts [GM23966 (healthy control) and GM03132 (DM1 with $(CTG)_{1700}$ repeats) for CTG repeats; GM23974 (healthy control) and GM09197 (HD with $(CAG)_{180}$ repeats) for CAG repeats; Coriell Institute for Medical Research] were seeded onto gelatin-coated culture plates (20,000–40,000 cells/well in 12-well plates) and cultured in DMEM supplemented with 10% FBS and 1× penicillin-streptomycin for 24 h. The cells were then transferred to a neuronal induction medium containing equal volumes of DMEM/F12 and Neurobasal Medium supplemented with 0.5% N-2, 1% B-27 (all from Gibco), and 100 μM cAMP (Sigma-Aldrich) with small molecules (0.5 mM valproic acid, 3 μM CHIR99021, 1 μM Repsox, 10 μM forskolin, 10 μM SP600125, 5 μM GO6983, 5 μM Y-27632, 20 μM ISX-9, and 2 μM I-BET151; Sigma-Aldrich) according to previously described methods (88, 89). Three days after treatment, we confirmed that a significant fraction (approximately 90%) of cells derived from healthy controls and DM1 patients exhibited typical neuronal morphology and expressed the neuronal marker Tuj1. Because iNeurons were not efficiently obtained from HD patient-derived fibroblasts used in this study (approximately less than 1%), the cells from HD patients and the corresponding healthy controls were used as fibroblasts for the following experiments.

Cell viability assay

Cell viability was measured using the Cell Counting Kit-8 (Dojindo Molecular Technologies, Inc.), according to the manufacturer's instructions. Neuro-2a cells were cultured in 96-well plates (2,000 cells/well) at 37°C for 24 h and treated with 0.1% DMSO (vehicle) or CWG-cPIP at different concentrations (0.1, 0.3, 1, 3, 10, or 30 μM). After 47 h, CCK-8 solution was added to each well, followed by incubation for 1 hour at 37°C. Absorbance at 450 nm was measured using a plate reader (Multiskan FC; Thermo Fisher Scientific). The viability of CWG-cPIP-treated cells was expressed as a percentage of that of the vehicle-treated cells.

RT-qPCR analysis

Sample preparation for RT-qPCR from Neuro-2a cells was performed using the SuperPrep II Cell Lysis & RT Kit qPCR (TOYOBO). Sample preparation for RT-qPCR from primary cultured neurons was performed using an RNeasy Mini Kit (QIAGEN) and PrimeScript RT Master Mix (Takara Bio, Inc.). RT-qPCR was performed using the KOD SYBR qPCR Mix (TOYOBO) on a CFX Connect Real-Time PCR System (Bio-Rad Laboratories, Inc.). Gene expression was assessed using differences in the normalized Ct (cycle threshold; $^{\Delta\Delta}$Ct) method after normalization to *Egfp* expression. Fold-changes were calculated using the $2^{-\Delta\Delta Ct}$ method. The following primers were used for RT-qPCR: *Egfp* (forward, 5′-CACATGAAGCAGCACGACTTC-3′; reverse, 5′-TTCAGCTCGATGCGGTTCAC-3′), HaloTag (forward, 5′-AGAATACATGGACTGGCTGC-3′; reverse, 5′-TCTTGCAGCAGATTCAGACC-3′),

mouse *Htt* (forward, 5′-CCCCATTCATTGCCTTGCTG-3′; reverse, 5′-CTTGAGCGACTCGAAAGCCT-3′), human *HTT* (forward, 5′-AGGTTCGCTTTTACCTGCGG-3′; reverse, 5′-CATCAGCTTTTCCAGGGTCG-3′), and *Gapdh* (forward, 5′-AACTTTGGCATTGTGGAAGG-3′; reverse, 5′-ACACATTGGGGGTAGGAACA-3′).

Antibodies

The following primary antibodies were used: anti-GFP (1:1000; ab290, Abcam), anti-GFP (1:500; clone 9F9.F9, ab1218, Abcam), anti-NeuN (1:2000; ABN90, Millipore), anti-cleaved caspase-3 (1:500; ab2302, Abcam), anti-Tuj1 (1:2000, 802001, BioLegend), anti-MBNL1 (1:500; ab45899, Abcam), anti-β-actin (1:1000; ab8227, Abcam), anti-Huntingtin (1:100; clone 3E10, sc-47757, Santa Cruz), anti-polyglutamine-expansion diseases marker (1:1000; clone 1C2, MAB1574, Millipore), anti-Huntingtin (1:500; clone MW8, MW8, DSHB deposited by Dr. P. H. Patterson), and anti-K63-specific ubiquitin (1:500; clone Apu3, 05-1308, Millipore). The following secondary antibodies were used: HRP-conjugated anti-mouse IgG antibody (1:5000; 1031-05, SouthernBiotech), and HRP-conjugated anti-rabbit IgG antibody (1:5000; 4050-05, SouthernBiotech), Alexa 488-conjugated donkey anti-rabbit (1:500; A-21206, Invitrogen), Alexa 594-conjugated donkey anti-rabbit (1:500; A-21207, Invitrogen), Alexa 488-conjugated donkey anti-mouse (1:500; A-21202, Invitrogen), Alexa 594-conjugated donkey anti-mouse (1:500; A-21203, Invitrogen), and Alexa 594-conjugated donkey anti-guinea pig (1:500; 706-585-148, Jackson ImmunoResearch Laboratories).

Histology

Brain tissues were fixed in 4% paraformaldehyde in PBS, sliced coronally at a thickness of 50 μm, and then incubated with 0.1% cresyl violet acetate (pH 4.8) for 10 min at 37°C. After differentiation with 95% ethanol and 0.1% acetate, the sections were dehydrated through a graded ethanol series, cleared with xylene, and mounted with Entellan new (Sigma-Aldrich). The sections were analyzed and imaged using a confocal laser scanning microscope (TCS SP8; Leica Microsystems).

Immunocytochemistry and immunohistochemistry

Immunocytochemistry and immunohistochemistry were performed as previously described (87). Briefly, brain slices and cells were fixed in 4% paraformaldehyde in PBS and then treated with PBS containing 0.3% Triton X-100 for 10 min. To detect polyQ-positive aggregates, immunofluorescence was performed as previously described (90). Briefly, the slices were treated with 88% formic acid for 10 min at room temperature and washed with running water, and then with PBS. The sections were then treated with PBS containing 0.4% Triton X-100 thrice for 10, 30, and 10 min. The samples were incubated overnight at 4°C with primary antibodies, washed in PBS, and incubated with fluorophore-labeled secondary antibodies. Nuclei were counterstained with DAPI (Thermo Fisher Scientific). The samples were mounted using VECTASHIELD (Vector Laboratories, Inc.), and fluorescence images were analyzed using a confocal laser scanning microscope (LSM900; Carl Zeiss).

FISH

Fixed brain slices and cells were washed three times with diethylpyrocarbonate-treated PBS (DPEC-PBS) for 10 min each and then incubated with 0.3% Triton X-100 in DPEC-PBS for 10 min. After several washes, the slices and cells were prehybridized with 40% formamide in 2× SSC (300 mM NaCl and 30 mM sodium citrate) for 10 min at room temperature, followed by incubation with a 1 nM Cy5-$(CAG)_{10}$ DNA probe in hybridization solution (2× SSC, 40% formamide, 10% dextran sulfate, 2 mM ribonucleoside-vanadyl complex, 0.5 mg/mL yeast transfer RNA) at 37°C overnight. After hybridization, the samples were washed with 40% formamide in 2× SSC and then with 1× SSC each for 15 min at 37°C. The samples were rinsed with DEPC-PBS and subjected to immunofluorescence procedure.

Western blotting

Immunoblotting was performed as described previously (87). Briefly, the cells were homogenized in RIPA buffer containing protease and phosphatase inhibitor cocktails (Nacalai Tesque, Inc.). Equivalent amounts of protein were subjected to SDS-PAGE. Separated proteins were transferred to an Immobilon PVDF membrane. The membrane was blocked with Tris-buffered saline (50 mM Tris-HCl, pH 7.5 and 150 mM NaCl) with 0.1% Tween 20 (TBST) solution containing 5% fat-free milk powder for 1 h at room temperature, and then incubated overnight at 4°C with primary antibodies. The membrane was then washed with TBST and incubated with HRP-conjugated secondary antibodies diluted in TBST for 1 h at room temperature. Blots were developed using an HRP substrate (32132; Thermo Fisher Scientific), and the immunoreactive bands were visualized using a chemiluminescence imaging system (FUSION SOLO; Vilber Bio Imaging).

RNA-Seq analysis

Total RNA was extracted from the mouse hippocampus using an RNeasy Mini Kit (QIAGEN). DNA libraries were prepared using NEBNext Ultra II Directional RNA Library Prep Kit for Illumina and sequenced by NextSeq 500 (Illumina, Inc.) to obtain single-end reads (75 nt) for off-target analysis and paired-end reads (150 nt) for splicing analysis, respectively. For off-target analysis, the extracted RNA was mixed with ERCC RNA Spike-In Mix (Invitrogen) containing 92 polyadenylated transcripts with concentration spanning 10^6-fold range prior to library preparation. After base calling, the sequences were demultiplexed and FASTQ files were generated using the Generate FASTQ Analysis Module in the Local Run Manager (Illumina, Inc.). The adapter sequence and low-quality ends were trimmed using the Trim Galore! (version 0.6.6). The RSEM package (version 1.3.3) in conjunction with the STAR aligner (version 2.7.9a) was used to align sequences with the mouse reference genome (UCSC GRCm38/mm10) and determine gene expression. Gene expression for each sample was further processed using DESeq2 (version 1.36.0) and expressed as normalized counts in a regularized logarithm (rlog). Expression levels for off-target analysis were normalized with those of spike-in controls. A list of genes containing non-pathological CWG repeats sequences was derived from the spliced RNA in the mouse reference genome (UCSC GRCm39/mm39). Genes with rlog-transformed expression levels were processed using DEGreport (version 1.32.0) for clustering analysis and further processed using clusterProfiler (version 4.4.4) for overrepresentation analysis. Alternative splicing events were quantified using rMATS (version 4.1.2).

Electrophysiology

To evaluate neuronal plasticity, hippocampal sections were prepared as previously described (87). Briefly, the brains were quickly removed from ether-anesthetized mice and chilled in ice-cold oxygenated artificial cerebrospinal fluid (124 mM NaCl, 5 mM KCl, 26 mM $NaHCO_3$, 2 mM $CaCl_2$, 2 mM $MgSO_4$, 1.25 mM NaH_2PO_4, and 10 mM D-glucose). Sagittal hippocampal slices of 400-μm thickness were transferred to a recording chamber, where they were allowed to recover for at least 1 h at room temperature (24°C to 26°C) before recording. A concentric bipolar stimulating electrode (FHC, Inc.) was placed in the stratum radiatum of CA1 to stimulate the Schaffer collateral pathway. An HFS of 100 Hz with a 1-s duration was applied twice with a 20-s interval. Traces were obtained and analyzed using SutterPatch version 2.2 (Sutter Instrument).

AAV preparation

Recombinant AAV9 particles were generated by co-transfection of AAVpro 293T cells (Takara Bio, Inc.) with three plasmids: pAAV (pAAV-CUG10, pAAV-CUG300, pAAV-Q23 or pAAV-Q74), pHelper (Stratagene), and pAAV2/9 (kindly provided by Dr. J. M. Wilson). The viral particles were harvested and purified using AAVpro Purification Kit Maxi (Takara Bio, Inc.) according to the manufacturer's instructions. Viral titers were measured using an AAVpro titration kit (Takara Bio, Inc.). For stereotaxic injection of these viruses into the mouse hippocampus, each virus was diluted to the same titer of 1.0×10^{13} vector genomes/mL.

Assay for interference with AAV infection

To evaluate the effect of CWG-cPIP on the stability of recombinant AAV, CWG-cPIP (0.756 nmol) was mixed with purified AAV (1.0×10^{10} vg) for 3 days at room temperature. The samples were subjected to SDS-PAGE and AAV capsid proteins VP1, VP2, and VP3 were visualized by Coomassie Brilliant Blue staining. Images were acquired using a chemiluminescence imaging system (FUSION SOLO), and the band intensities were normalized by values of vehicle-treated AAV for each repeat length. Transduction efficiency was evaluated by AAV infection in HEK293 cells. Twenty-four hours after cell seeding (20,000 cells/well in 12-well plates), recombinant AAV (multiplicity of infection: 1×10^5 vg/cell) and CWG-cPIP (0.756 nmol) were co-treated and cultured for another 3 days. Cells were fixed, stained with DAPI, and subjected to confocal microscopy (LSM900). GFP-positive cells were considered as AAV-infected populations.

Stereotaxic surgery

Male mice were stereotaxically injected with CWG-cPIP and recombinant AAV9 at nine weeks of age. Under anesthesia, the mice were placed in a stereotaxic instrument (Narishige), and holes were drilled in the cranium. A mixture of CWG-cPIP (0.5 μL, 0.756 nmol) and each AAV9 (1.0 μL, 1.0×10^{13} vector genomes/mL) in 1.5% DMSO/PBS per hemisphere was injected bilaterally into the CA1 region of the dorsal hippocampus using a 26s-gauge needle. Coordinates relative to the bregma were as follows (in mm): anterior, -2.2; lateral, ±1.5; ventral, -2.1 for hippocampus; anterior, -0.5; lateral, ±1.0; ventral, -2.3 for

lateral ventricle. Three weeks after the injection, these mice were behaviorally, electrophysiologically, and immunohistochemically analyzed. To assess tissue distribution and retention of injected CWG-cPIP, FITC-labeled CWG-cPIP (0.5μL in 1.5 or 10% DMSO/PBS per hemisphere) was injected at doses of 0.00756, 0.0756, 0.756, or 6.048 nmol per hemisphere, and immunohistochemical analysis was performed 1, 3, and 7 days later.

Behavioral analysis

Mice injected with AAVs were subjected to the Y-maze, NOR, PA tests, which were prepared as previously described (87). In the Y-maze test, spontaneous alternation behavior in the Y-maze was assessed as a spatial reference memory task. The apparatus consisted of three identical Plexiglas arms ($44 \times 13 \times 12$ cm^3). Mice were placed at the end of one arm and were allowed to move freely through the maze during an 8-min session. The sequence of arm entries was recorded manually. Alternation was defined as entry into all three arms on consecutive choices. The maximum number of alternations was defined as the total number of arms entered minus two, and the percentage of alternations was calculated as the actual alternations/maximum alternations × 100. The total number of arms entered during each session was determined. In the NOR test, mice were individually habituated to an open-field box ($28 \times 17 \times 13$ cm^3) for 2 consecutive days. During the acquisition phase, two objects of the same material were placed symmetrically at the center of the box for 10 min. Twenty-four hours later, one object was replaced by a novel object, and exploratory behavior was analyzed again for 10 min. After each session, the objects were thoroughly cleaned with 70% ethanol to prevent odor recognition. Exploration of an object was defined as rearing on the

object, sniffing it at a distance of < 1 cm, touching it with the nose, or both. Successful recognition was reflected by preferential exploration of the novel object. The discrimination of o novelty was assessed by comparing the difference between exploratory contacts of novel and familiar objects and the total number of contacts with both, making it possible to adjust for differences in total exploration contacts. In the PA test, training and retention trials were conducted in a box consisting of dark and light compartments (13 × 11 × 20 cm^3). The floor was constructed with stainless steel rods, and the rods in the dark compartment were connected to an electronic stimulator (Med Associates, Inc.). Mice were habituated to the apparatus for 2 days prior to passive avoidance acquisition. During training, a mouse was placed in the light compartment, and on entering the dark compartment, the door was closed and an electric shock (0.5 mA for 3 s) was delivered from the floor. The mouse was removed from the apparatus 30 s later. The next day, each mouse was placed in the light compartment, and step-through latency was recorded for over 300 s to assess retention. The videotapes for all behavioral analyses were scored by a trained observer blinded to the drug treatment.

Motor function in R6/2 mice were assessed by rotarod and hind-limb clasping tests as previously described (91). In the rotarod test, mice were placed on a stationary rod (30 mm diameter; Muromachi Kikai) and left in place for 60 s. The mice were then forced to walk on the accelerating rods (4–40 rpm) for up to 300 s. An hour later, the mice were subjected to the second trial, and the latency to fall was measured for each trial. In the hind-limb clasping test, mice were suspended by their tails for 30 s at a height of 50 cm from the home-cage and hindlimb clasping was scored as follows: score 0, hind-limbs consistently stretched outward from the abdomen; score 1, hind-limbs individually, but not both at the

same time, retract toward the abdomen with a cumulative time less than 15 s; score 2, hind-limbs individually, but not both at the same time, retract toward the abdomen with a cumulative time 15 s or more; score 3, both hind-limbs retract toward and touch the abdomen at the same time.

Supplementary Figures

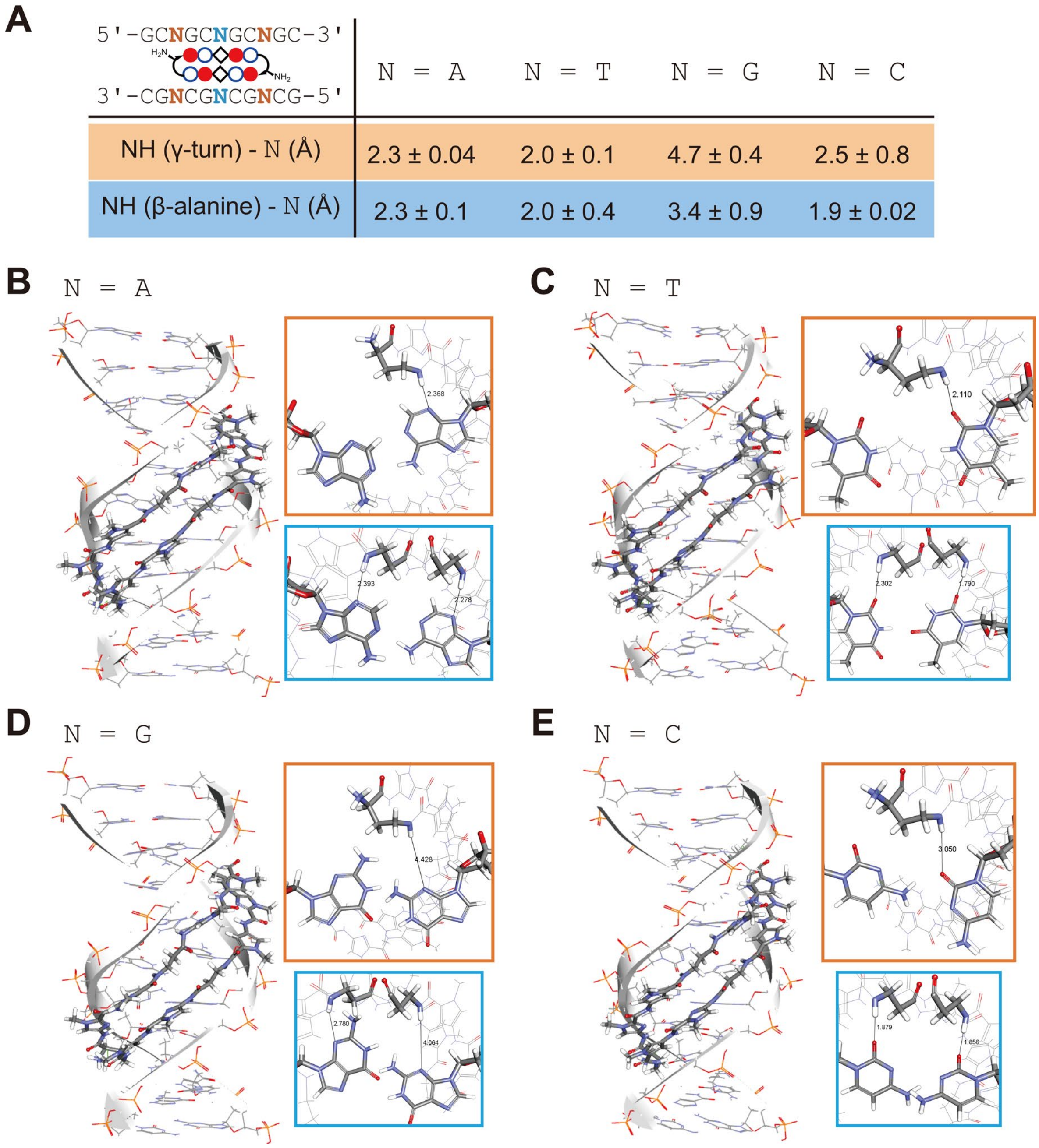

5'-GCNGCNGCNGC-3' / 3'-CGNCGNCGNCG-5'	N = A	N = T	N = G	N = C
NH (γ-turn) - N (Å)	2.3 ± 0.04	2.0 ± 0.1	4.7 ± 0.4	2.5 ± 0.8
NH (β-alanine) - N (Å)	2.3 ± 0.1	2.0 ± 0.4	3.4 ± 0.9	1.9 ± 0.02

Supplementary Figure 1. Binding modes of CWG-cPIP for CNG repeat DNA.

(A) The interaction distances of hydrogen bonds between DNA base pairs (N: A, T, G, or C) and γ-turn (orange) or β-alanine (blue) in CWG-cPIP. For description of a schematic illustration of DNA sequence recognition by CWG-cPIP, see Figure 1A. **(B–E)** Molecular models of CWG-cPIP/double-stranded CAG- (B), CTG- (C), CGG- (D), or CCG- (E) DNA complex by computer-assisted molecular simulation. Data represent mean ± SEM.

FITC-labeled CWG-cPIP

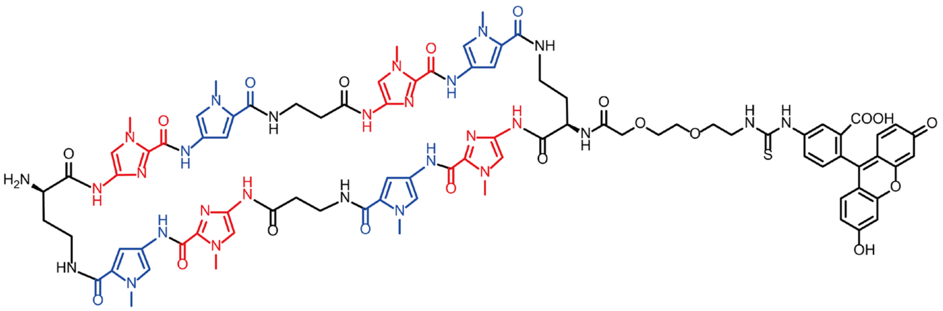

Chemical Formula: $C_{85}H_{92}N_{28}O_{20}S$
Exact Mass: 1856.68
Molecular Weight: 1857.91

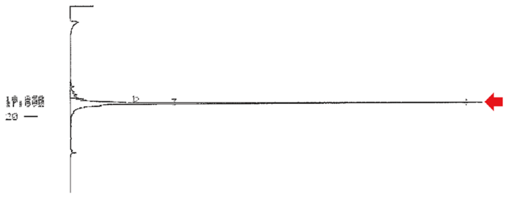

-- % CALCULATION RESULT --

WINDOW = 0 %   SCALE FACTOR = 1.0000   PEAK AREA

PEAK#	RT(min)	AREA	HEIGHT	MK	AREA%
1	16.850	386146	28580	V	2.5164
2	17.133	514862	34332	VS	3.3552
3	17.500	1711674	118647	VS	11.1544
4	17.692	12732563	1047508	EEE	82.9740
	TOTAL	15345245	1229068		100.0000

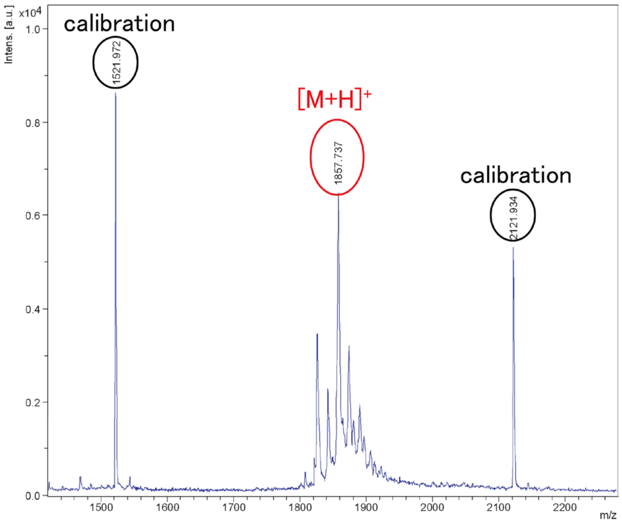

Supplementary Figure 2. Synthesis of FITC-labeled CWG-cPIP.

(top) Chemical structure of FITC-labeled CWG-cPIP. (bottom) HPLC and MALDI-TOF MS spectra of FITC-labeled CWG-cPIP. Conditions: equilibrated with 0.1% trifluoroacetic acid with a linear gradient from 0% to 100% acetonitrile at a flow rate of 1.0 mL/min for 40 min, detected at 254 nm. Arrows indicate the peak and the retention time (17.692 min). m/z found; 1857.737.

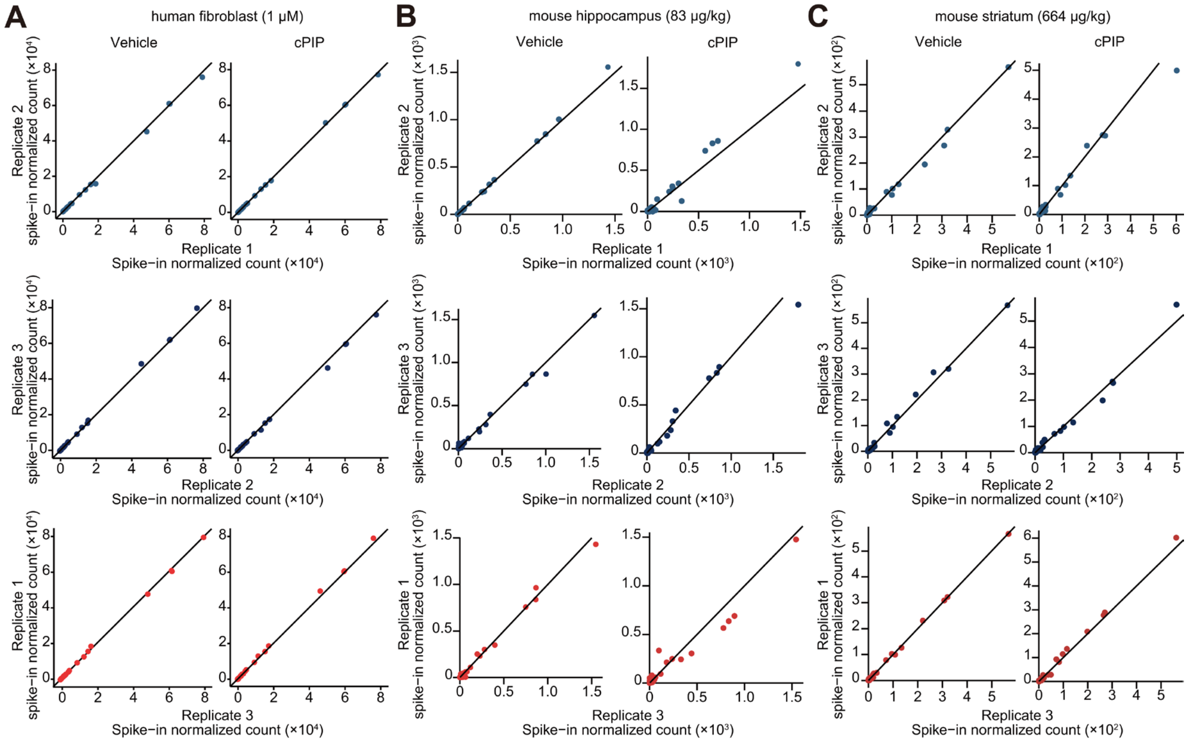

Supplementary Figure 3. Abundances of spike-in controls in RNA-seq analyses.

(A–C) Comparisons of transcripts with normalized count of spike-in controls between replicates in RNA-seq analyses using human fibroblasts treated with 1μM CWG-cPIP (A), mouse hippocampus with 83 μg/kg CWG-cPIP (B), and mouse striatum with 664 μg/kg CWG-cPIP (C).

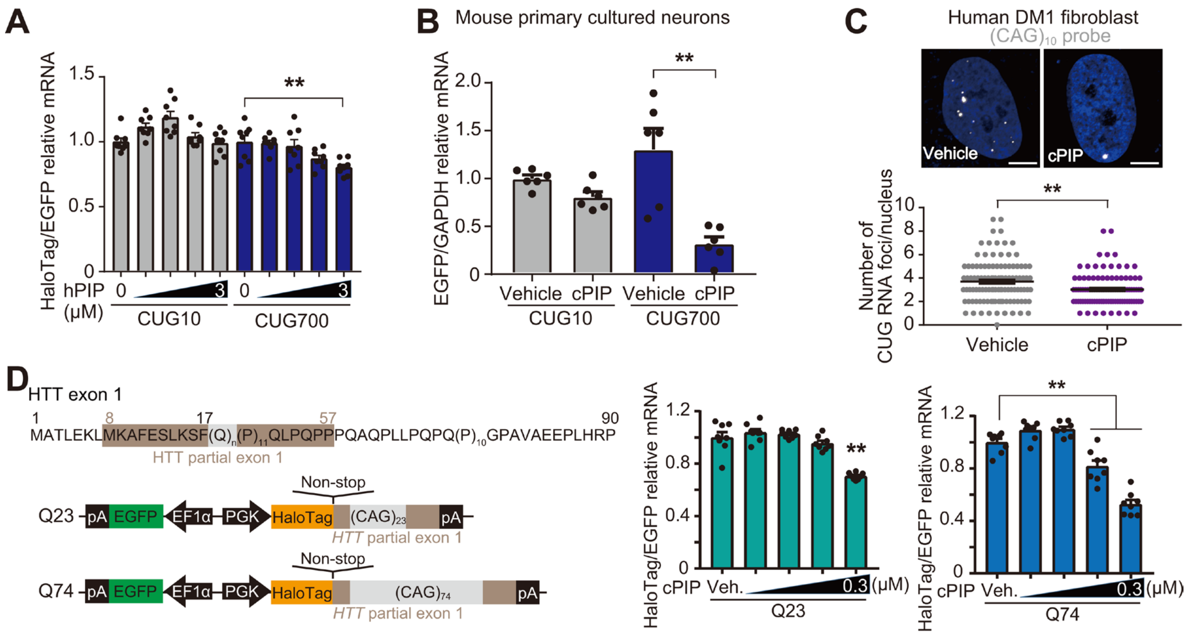

Supplementary Figure 4. Inhibition of transcription and pathogenic CUG RNA foci in CWG repeat-expanded cell models by CWG-cPIP treatment.

(A) Quantification of HaloTag mRNA levels in Neuro-2a cells treated with CWG-hPIP at concentrations of 0.1, 0.3, 1, and 3 μM. $^{**}P < 0.01$ by one-way ANOVA with Bonferroni's multiple comparisons test. n = 8 each. **(B)** Quantification of *Egfp* mRNA levels in mouse primary neurons treated with 1 μM CWG-cPIP. $^{**}P < 0.01$ by two-way ANOVA with Bonferroni's multiple comparisons test. n = 6 each. **(C)** Representative confocal images of CUG-RNA foci (white) in DM1 patient-derived fibroblasts (top). Scale bars, 5 μm; quantification of CUG-RNA foci (bottom). $^{**}P < 0.01$ by two-sided unpaired Student's t-test. Vehicle: n = 108 cells; CWG-cPIP: n = 100 cells. **(D)** Amino acid sequences of human HTT exon 1 (left, top). Residue numbers refer to HTT with Q23 repeat. Schematic representation of constructs with CAG repeat sequences in a coding region used for RT-qPCR in Neuro-2a cells (left, bottom); quantification of HaloTag mRNA levels (right).

CWG-cPIP concentrations were 0.01, 0.03, 0.1, and 0.3 μM. **$P < 0.01$ by one-way ANOVA with Bonferroni's multiple comparisons test. $n = 8$ each. Data represent mean ± SEM. Source data are provided in Supplementary File 6.

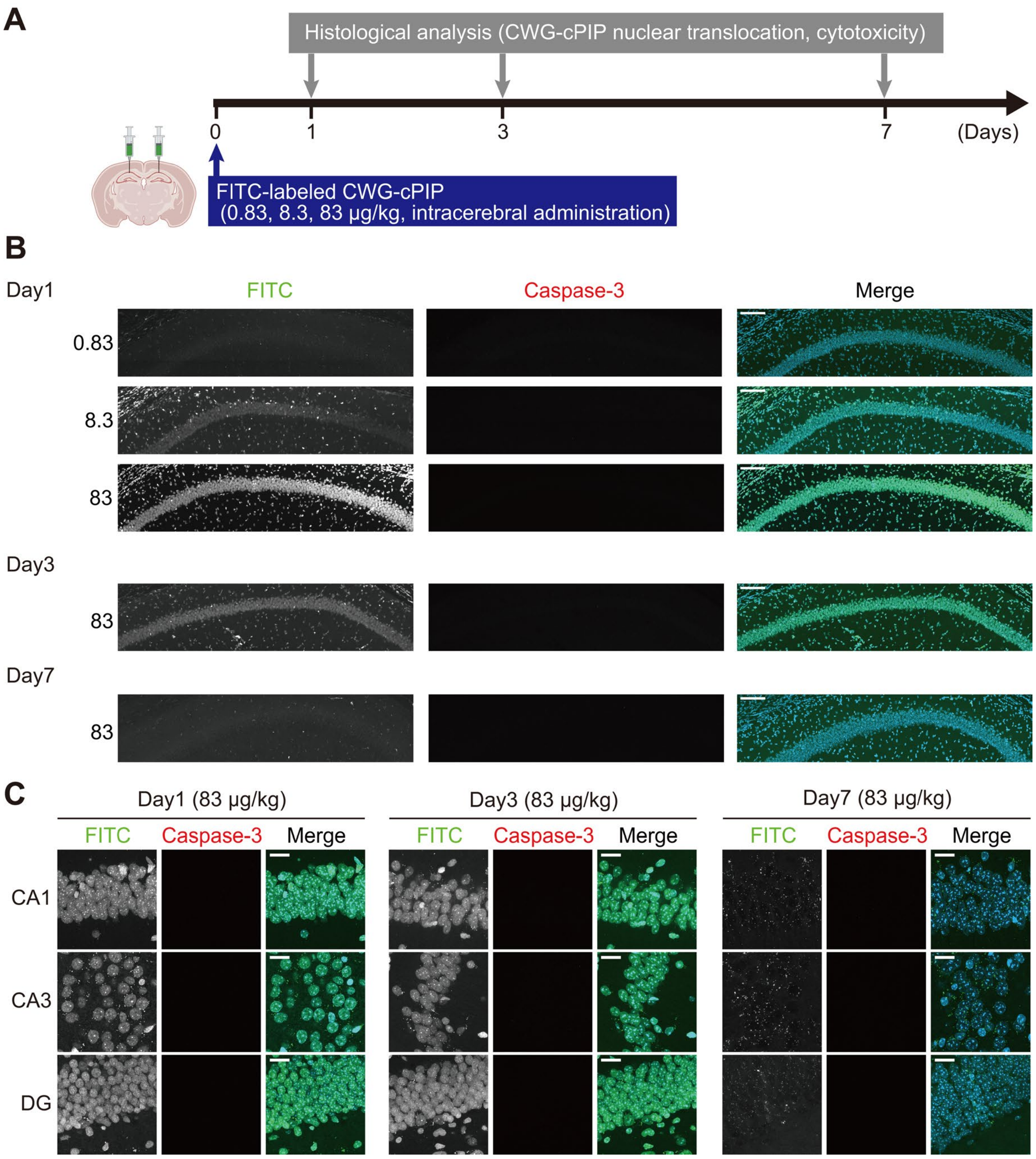

Supplementary Figure 5. Nuclear penetration of CWG-cPIP without cell toxicity after intracerebral injection.

(A) Experimental diagram of intracerebral injection of FITC-labeled CWG-cPIP into intact mice and the immunohistochemical analysis. **(B, C)** Representative confocal images of

FITC-labeled CWG-cPIP and cleaved caspase-3 in the hippocampal CA1, CA3, and DG subregions. Scale bars, 100 μm (B) and 20 μm (C).

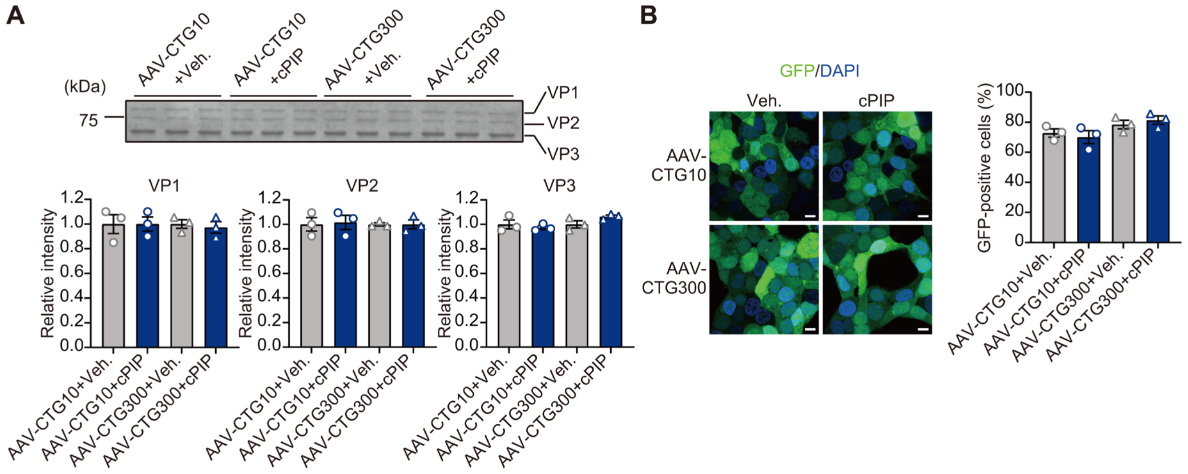

Supplementary Figure 6. No interference of CWG-cPIP on recombinant AAV.

(A) Gel images stained with Coomassie Brilliant Blue after treatment of recombinant AAV with CWG-cPIP in vitro (top) and quantifications of the band intensities (bottom). n = 3 each. Statistics were performed by two-sided unpaired Student's t-test. **(B)** Representative images of GFP-positive HEK293 cells co-treated with recombinant AAV and CWG-cPIP (left) and quantification of GFP-positive cells (right). n = 3 each, acquired from 3 images in each experiment. Statistics were performed by two-sided unpaired Student's t-test. Data represent mean ± SEM. Source data are provided in Supplementary File 6.

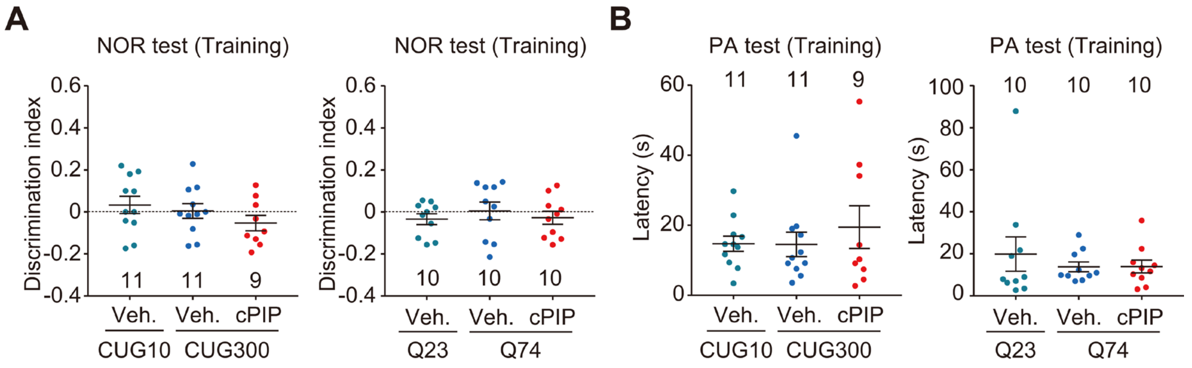

Supplementary Figure 7. Normal behaviors of mice in training sessions of memory-related tests.

(A) Discrimination indices in the training sessions of the NOR test. Statistics were performed by one-way ANOVA with Bonferroni's multiple comparisons test. CUG10 + vehicle and CUG300 + vehicle: n = 11 mice; CUG300 + CWG-cPIP: n = 9 mice (left); n = 10 mice each (right). **(B)** Latency to enter the dark compartment in the training sessions of the PA test. Statistics were performed by one-way ANOVA with Bonferroni's multiple comparisons test. CUG10 + vehicle and CUG300 + vehicle: n = 11 mice; CUG300 + CWG-cPIP: n = 9 mice (left); n = 10 mice each (right). Data represent mean ± SEM. Source data are provided in Supplementary File 6.

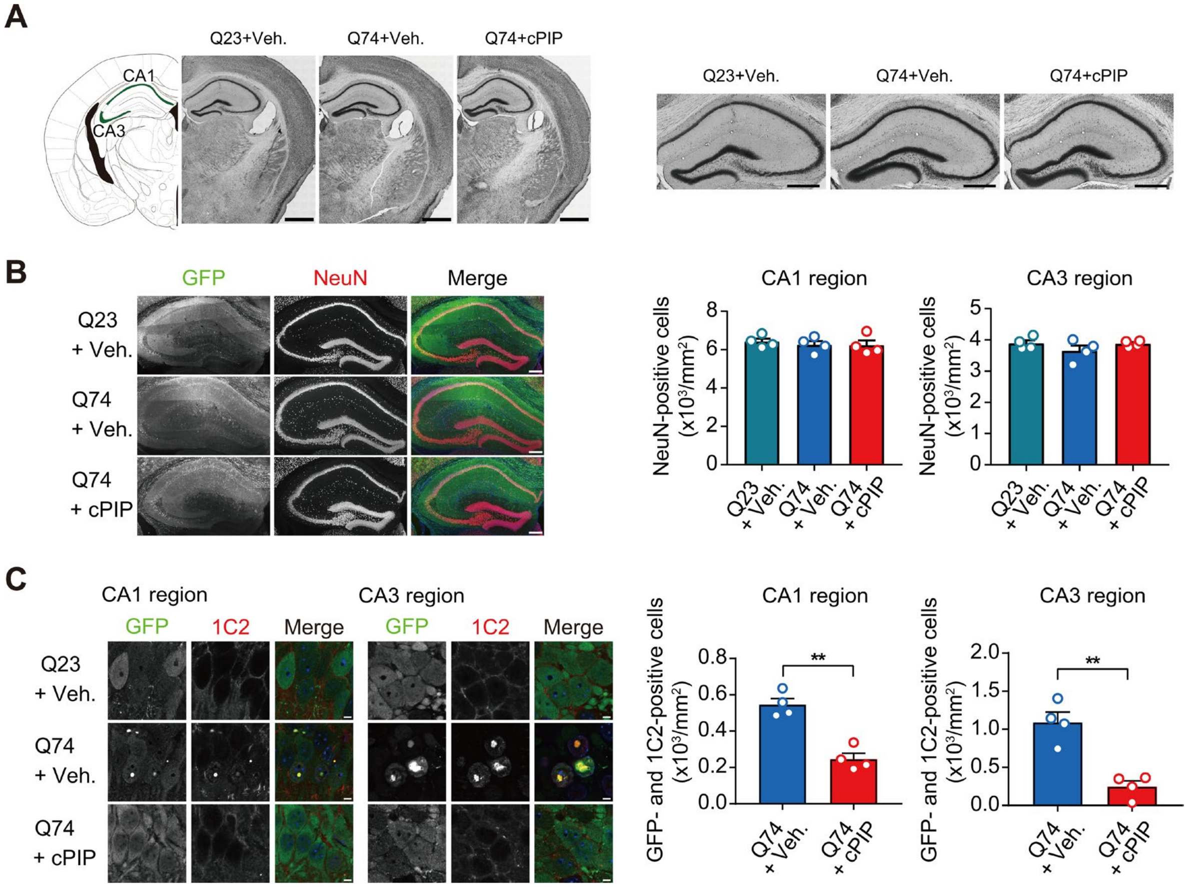

Supplementary Figure 8. Inhibition of polyQ aggregation seen in a CAG repeat-expanded mouse model by CWG-cPIP treatment.

(A) Representative confocal images of Nissl-stained sections. Scale bars, 1 mm (left) and 500 μm (right). **(B)** Representative confocal images of GFP (green) and NeuN (red) in the hippocampus (left) and the quantification of NeuN-positive cells in CA1 and CA3 regions (right). Statistics were performed by one-way ANOVA with Bonferroni's multiple comparisons test. *n* = 4 mice each, averaged from three independent replicates (three slices) per mouse. Scale bars, 200 μm. **(C)** Representative confocal images of polyQ aggregates in the hippocampal CA1 and CA3 regions (left) and their quantification (right). $^{**}P < 0.01$ by two-sided unpaired Student's t-test. *n* = 4 mice each, averaged from three independent

replicates (three slices) per mouse. Scale bars, 5 µm. Data represent mean ± SEM. Source data are provided in Supplementary File 6.

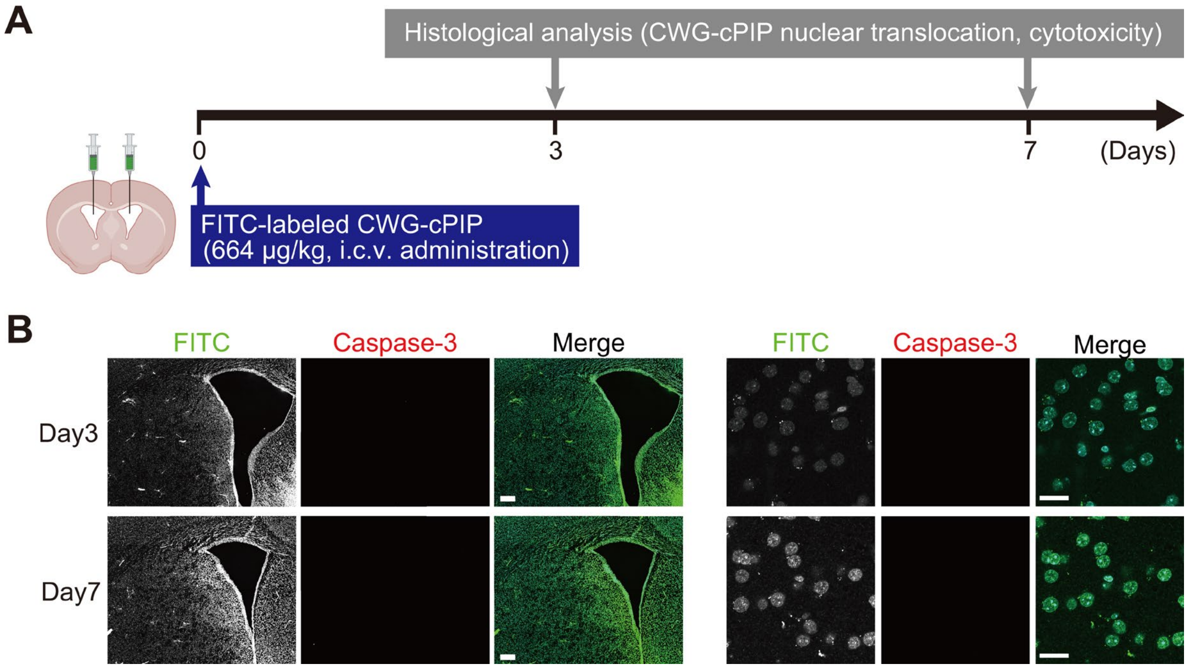

Supplementary Figure 9. Nuclear penetration of CWG-cPIP without cell toxicity after i.c.v. injection.

(A) Experimental diagram of i.c.v. injection of FITC-labeled CWG-cPIP into intact mice and the immunohistochemical analysis. **(B, C)** Representative confocal images of FITC-labeled CWG-cPIP and cleaved caspase-3 in the striatum. Scale bars, 200 μm (B) and 20 μm (C).

	double-stranded DNA 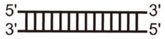				1bp mismatched hairpin DNA 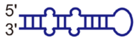				1bp mismatched hairpin RNA 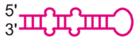	
	d(CAG/CTG)	d(CCG/CGG)	AT rich	GC rich	$d(CAG)_{10}$	$d(CTG)_{10}$	$d(CGG)_{10}$	$d(CCG)_{10}$	$r(CUG)_{10}$	$r(CAG)_{10}$
T_m (°C) (vehicle)	43.4 ± 0.1	57.6 ± 0.9	28.4 ± 3.6	79.1	49.9 ± 0.3	50.7 ± 0.1	66.8 ± 0.6	48.3 ± 0.1	47.8 ± 0.2	59.7 ± 0.5
ΔT_m (hPIP-vehicle)	38.8 ± 0.8	17.1 ± 2.4	10.8 ± 0.5	-11.6 ± 2.9	36.2 ± 0.05	34.5 ± 0.3	3.24 ± 0.6	31.1 ± 1.7	-0.13 ± 1.7	-2.70 ± 0.6
ΔT_m (cPIP-vehicle)	51.6 ± 0	14.5 ± 0.4	2.24 ± 0.04	-1.22 ± 1.3	45.1 ± 0	41.4 ± 0.4	3.64 ± 0.3	30.3 ± 0.6	-0.14 ± 0.1	-2.27 ± 0.2

Supplementary Table 1. T_m and ΔT_m in the melting temperature assay for indicated DNAs and RNAs with PIPs addition.

References

87. Shioda N, et al. Targeting G-quadruplex DNA as cognitive function therapy for ATR-X syndrome. *Nat. Med.* 2018;24(6):802–813.

88. Hu W, et al. Direct Conversion of Normal and Alzheimer's Disease Human Fibroblasts into Neuronal Cells by Small Molecules. *Cell Stem Cell* 2015;17(2):204–212.

89. Yang J, et al. Small molecular compounds efficiently convert human fibroblasts directly into neurons. *Mol. Med. Rep.* 2020;22(6):4763–4771.

90. Osmand AP, et al. Imaging Polyglutamine Deposits in Brain Tissue. *Methods Enzymol.* 2006;412:106–122.

91. Kumar MJV et al. Spatiotemporal analysis of soluble aggregates and autophagy markers in the R6/2 mouse model. Sci. Rep. 2021;11(1):96.
